# Novel insight into the reaction of nitro, nitroso and hydroxylamino benzothiazinones and of benzoxacinones with *Mycobacterium tuberculosis DprE1*

**DOI:** 10.1038/s41598-018-31316-6

**Published:** 2018-09-07

**Authors:** Adrian Richter, Ines Rudolph, Ute Möllmann, Kerstin Voigt, Chun-wa Chung, Onkar M. P. Singh, Michael Rees, Alfonso Mendoza-Losana, Robert Bates, Lluís Ballell, Sarah Batt, Natacha Veerapen, Klaus Fütterer, Gurdyal Besra, Peter Imming, Argyrides Argyrou

**Affiliations:** 1 Institut für Pharmazie, Martin-Luther-Universität Halle-Wittenberg, Wolfgang-Langenbeck-Str. 4, 06120 Halle (Saale), Germany; 2 Leibniz-Institut für Naturstoff-Forschung und Infektionsbiologie - Hans-Knöll-Institut, Beutenbergstrasse 11a, 07745 Jena, Germany; 3 Platform Technology & Science, GlaxoSmithKline, Gunnels Wood Road, Stevenage, SG1 2NY United Kingdom; 4 Diseases of the Developing World, Tres Cantos Medicines Development Campus, GlaxoSmithKline, Severo Ochoa 2, 28760 Tres Cantos, Madrid, Spain; 5 School of Biosciences, University of Birmingham, Edgbaston Birmingham, B15 2TT United Kingdom; 6 Present Address: Discovery Biology, Discovery Sciences, IMED Biotech unit, AstraZeneca, Cambridge, United Kingdom

## Abstract

Nitro-substituted 1,3-benzothiazinones (nitro-BTZs) are mechanism-based covalent inhibitors of *Mycobacterium tuberculosis* decaprenylphosphoryl-β-D-ribose-2′-oxidase (DprE1) with strong antimycobacterial properties. We prepared a number of oxidized and reduced forms of nitro-BTZs to probe the mechanism of inactivation of the enzyme and to identify opportunities for further chemistry. The kinetics of inactivation of DprE1 was examined using an enzymatic assay that monitored reaction progress up to 100 min, permitting compound ranking according to *k*_inact_/*K*_i_ values. The side-chain at the 2-position and heteroatom identity at the 1-position of the BTZs were found to be important for inhibitory activity. We obtained crystal structures with several compounds covalently bound. The data suggest that steps upstream from the covalent end-points are likely the key determinants of potency and reactivity. The results of protein mass spectrometry using a 7-chloro-nitro-BTZ suggest that nucleophilic reactions at the 7-position do not operate and support a previously proposed mechanism in which BTZ activation by a reduced flavin intermediate is required. Unexpectedly, a hydroxylamino-BTZ showed time-dependent inhibition and mass spectrometry corroborated that this hydroxylamino-BTZ is a mechanism-based suicide inhibitor of DprE1. With this BTZ derivative, we propose a new covalent mechanism of inhibition of DprE1 that takes advantage of the oxidation cycle of the enzyme.

## Introduction

*Mycobacterium tuberculosis* (Mtb) remains the top bacterial killer worldwide, often helped in its consumptive effect by AIDS^1^. New antitubercular agents are needed that, in combination with other drugs, decrease the duration of treatment and also address multi- and extensively-drug resistant forms of Mtb (MDR, XDR). This goal can only be achieved through the discovery and development of novel drug substances that operate via hitherto not addressed targets^2^. Decaprenylphosphoryl-β-D-ribose-2′-oxidase (DprE1) has been shown to meet these requirements^3^ after confirmation of DprE1 as the target of new potent and selective antitubercular benzothiazinones (BTZs)^4^.

**Figure 1:**
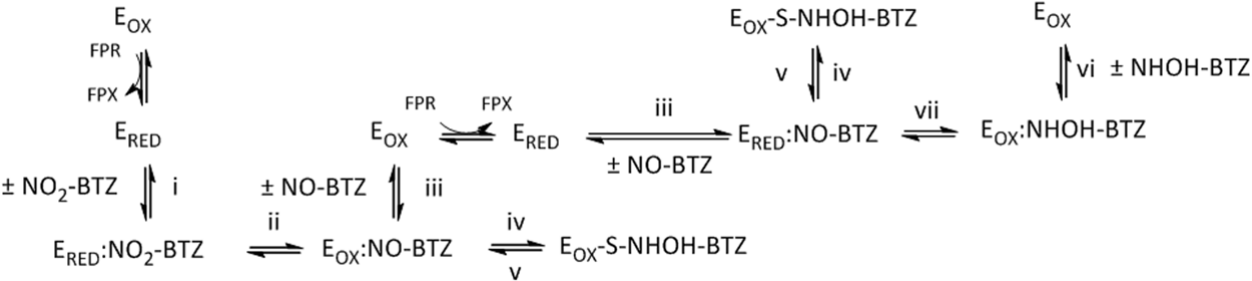
Mechanism of inhibition of DprE1 by BTZs. (i) noncovalent recognition of nitro(NO_2_)-BTZs by DprE1; (ii) reduction of nitro-BTZs to nitroso(NO)-BTZs by DprE1; (iii) release of nitroso-BTZs; (iv) covalent binding of nitroso-BTZs to Cys387; (v) cleavage of the bond between BTZs and DprE1; (vi) noncovalent recognition of hydroxylamino(NHOH)-BTZs by DprE1; (vii) oxidation of the hydroxylamino-BTZs to nitroso-BTZs by DprE1.

DprE1 is the first of two enzymes that catalyze the conversion of decaprenylphosphoryl-β-D-ribose (DPR) to decaprenylphosphoryl-β-D-arabinose (DPA)^5^. Arabinose polymers form a large fraction and essential part of the mycobacterial cell wall. The DPR to DPA conversion appears to be the sole source of D-arabinofuranosyl residues in Mtb. In its natural context, DprE1 catalyzes the oxidation of the 2′ hydroxyl group of DPR to give the ketone decaprenylphosphoryl-2-keto-β-D-erythropentofuranose (DPX), with flavin adenine dinucleotide (FAD) acting as the oxidant in the reaction. While BTZs have been defined as mechanism-based inhibitors, it is worth noting that they do not operate through the forward physiological mechanism of DprE1, but its reverse function. After BTZs have entered the catalytic pocket of DprE1 containing the reduced form of the flavin cofactor (FADH_2_; see Fig. 1), which was generated by oxidation of the substrate, the nitro group of the BTZs is reduced to nitroso which can then form a covalent bond with a nearby cysteine residue (Cys387, Mtb enzyme numbering)^6,7^. Cys387 is not essential for DprE1 activity as shown by site directed mutagenesis^6,8^ and by the fact that Mtb strains with Cys387 residues mutated to Ser or Gly are viable^4^. Therefore, BTZs can covalently inhibit DprE1 only in the presence of its natural substrate or close analogues, e.g. farnesylphosphoryl-β-D-ribofuranose (FPR) that is often used in enzymatic assays because reductive activation of the nitro to the nitroso form of the BTZs is mediated by enzyme-bound FADH_2_^9,10^.

Whilst the BTZs have been studied extensively and significant progress towards understanding their mechanism of action has been made, there is a significant gap in our understanding of the enzymatic and cellular potencies of these compounds. Previous studies have shown a wide range in cellular potencies as measured by mycobacterial minimum inhibitory concentration (MIC)^4,11,12^. In the single study for which enzymatic IC_50_ values on the enzyme have additionally been measured, for BTZ043 the IC_50_ is much higher than the MIC value^9^. In this paper, we present an extensive study of DprE1 inhibition kinetics by known and new BTZs in order to bridge the gap between enzymatic and cellular data and possibly identify new BTZ leads. In addition, we synthesized a novel BTZ, two nitroso- and one hydroxylamino-containing compound (see Results) in order to further probe the mechanism of inactivation of DprE1 through the combined used of enzyme inhibition kinetics, mass spectrometry and X-ray crystallography.

## Results

### A panel of antimycobacterial BTZs and BOZs

A new synthetic method for the BTZs was developed^13^, in which the sulfur and nitrogen of the benzothiazinone scaffold are incorporated in a single step. This was accomplished by the use of asymmetrically substituted thiourea reagents (synthesis details can be found in Supplemental Methods).

Table 1 lists the compounds that were synthesized for the present study. The reader is referred to the generic structure and numbering of the molecule at the top of Table 1. Compounds 7^12^, 6^12^ and 9 represent a series that have the same benzothiazinone scaffold but differ in the cyclic group attached to the 2-position, namely piperidine, morpholine, and dimethylpiperidine, respectively. Compound 11, the first 7-substituted BTZ to be tested, will be described below. Compounds 4, 3 and 5 are the benzoxacinone (BOZ) analogues of 7, 6 and 9, respectively. The BOZs have an oxygen atom instead of a sulfur at the 1-position of the molecule and these were synthesized in order to probe the potency of the molecules relative to the BTZ counterparts. Compounds 12 (BTZ043^4^) and 13 (PBTZ169^14^) are the most potent BTZs reported to date and 13 in particular shows great promise for further development. We have synthesized compound 10, which is the BOZ equivalent of 13, in order to further probe the effect on potency of the sulfur to oxygen heteroatom substitution at the 1-position of the molecule.

**Table 1:** Correlation between the kinetics of inactivation of Mtb-DprE1 with cellular potency by BTZs and BOZs.


**A series of Nitrobenzothiazinones (Nitro-BTZs)**				
				
*k*_inact_/*K*_i_ (M^−1^ s^−1^)	39 ± 2	17 ± 2	240 ± 16	340 ± 50
CLND Solubility (μM)	370	≥450	90	11
MIC *Mtb*-H37Rv (n = 3)	3.3 μM 1.2 μg/ml	5.9 μM 2.1 μg/ml	0.8 μM 0.31 μg/ml	1.6 μM 0.6 μg/ml
MIC *M. vaccae* 10670 (n = 1)	1.1 μM 0.4 μg/ml	8.6 μM 3.1 μg/ml	<0.13 μM <0.05 μg/ml	0.5 μM 0.2 μg/ml
**A series of Nitrobenzoxacinone (Nitro-BOZs) analogues of the Nitro-BTZs (above)**				
				
*k*_inact_/*K*_i_ (M^−1^ s^−1^)	5.6 ± 0.1	4.2 ± 0.1	7.7 ± 0.3	
CLND Solubility (μM)	≥510	≥510	130	
MIC *Mtb*-H37Rv (n = 3)	16 μM 5.3 μg/ml	3.9 μM 1.4 μg/ml	6.5 μM 2.4 μg/ml	
MIC *M. vaccae* 10670 (n = 1)	4.6 μM 1.6 μg/ml	18 μM 6.2 μg/ml	0.5 μM 0.2 μg/ml	
**The most potent Nitro-BTZs and Nitro-BOZ**				
				
*k*_inact_/*K*_i_ (M^−1^ s^−1^)	720 ± 20	N.A.^c^	300 ± 40	
CLND Solubility (μM)	32	32	12	
MIC *Mtb*-H37Rv (n = 3)	2.3 nM 1 ng/ml	0.42 nM 0.19 ng/ml	310 nM 0.14 μg/ml	
MIC *M. vaccae* 10670 (n = 1)	1 nM 0.4 ng/ml	N.D.	<110 nM <0.05 μg/ml	
**Reduced forms of nitro-BTZs**				
				
Redox status relative to Nitro-BTZs	2-electron reduced	4-electron reduced	2-electron reduced	
*k*_inact_/*K*_i_ (M^−1^ s^−1^)	N.A.^a^	N.A.^b^	39 ± 1	
CLND Solubility (μM)	250	10	210	
*Mtb*-H37Rv MIC (n = 3)	82% Inhibition at 80 μM	>80 μM	67% Inhibition at 0.16 μM	
*M. vaccae* 10670 MIC (n = 1)	73 μM 25 μg/ml	18 μM 6.2 μg/ml	0.06 μM 0.03 μg/ml	

^a^*k*_inact_/*K*_i_ value for 1 could not be obtained because the time-courses only mildly deviate from linearity in the first 100 min of reaction making fitting to Eq. 1 difficult; the IC_50_ decreases from 5 to 3 μM during the first 100 min of reaction (see Fig. S1A).

^b^*k*_inact_/*K*_i_ value for 2 could not be obtained because the *k*_obs_ versus inhibitor concentration relationship was concave up; the IC_50_ decreases from 3.5 to 0.8 μM during the first 100 min of reaction.

^c^*k*_inact_/*K*_i_ value for 13 could not be obtained using the present assay due to tight-binding limit considerations^19^; the 10–170 nM inhibitor concentration range where inhibition is observed is less than or equal to the concentration of DprE1 in the assay resulting in inhibitor depletion over time (see Fig. S1A).

Several other compounds were also synthesized to probe the mechanism of inhibition of DprE1 by the BTZs. Compounds 1 and 8 are the two-electron reduced forms of 7 and 12, respectively, also referred to as nitroso. These are intermediates that are proposed to be generated in the DprE1 active site as the BTZs are reduced by the reduced flavin cofactor (FADH_2_) in the first step of the mechanism of inactivation of the enzyme by the BTZs^6,7,15^ (see Introduction and Fig. 1). We were interested to compare the kinetics of inactivation of DprE1 by 1 and 8, which do not require activation, relative to the BTZ counterparts, 7 and 12, that require reductive activation. It is worth noting that the synthetic pathway^16^ for the nitroso-BTZs such as 8 has not been described before (see Supplemental Methods). Compound 2 is the four-electron reduced form of 7, also referred to as hydroxylamino-BTZ, and was synthesized as a potential noncovalent inhibitor control. However, it turned out to be a novel mechanism-based suicide substrate (see later). Finally, compound 11 was designed to probe two distinct chemical mechanisms of covalent bond formation with the enzyme that have been proposed previously. The first mechanism was proposed by Trefzer *et al*.^6,7^, where reductive activation of the BTZ by the reduced flavin intermediate of DprE1 is followed by covalent bond formation with the nitroso intermediate (see Introduction and Fig. 1). The second mechanism was proposed by Tiwari *et al*. and it involves nucleophilic substitution at the 7-position of the BTZ (see later)^17^.

### Kinetics of inactivation of DprE1

As mentioned in “Introduction”, BTZs have a wide range of MIC values^4,11,12^. Enzymatic IC_50_ values have only heen measured in a single study^9^ and for BTZ043 this value is much higher than the MIC. To gain insight into the apparent disconnect between enzymatic and cellular data, we performed mechanism of inhibition studies using purified recombinant Mtb-DprE1 enzyme on the compounds shown in Table 1.

**Figure 2:**
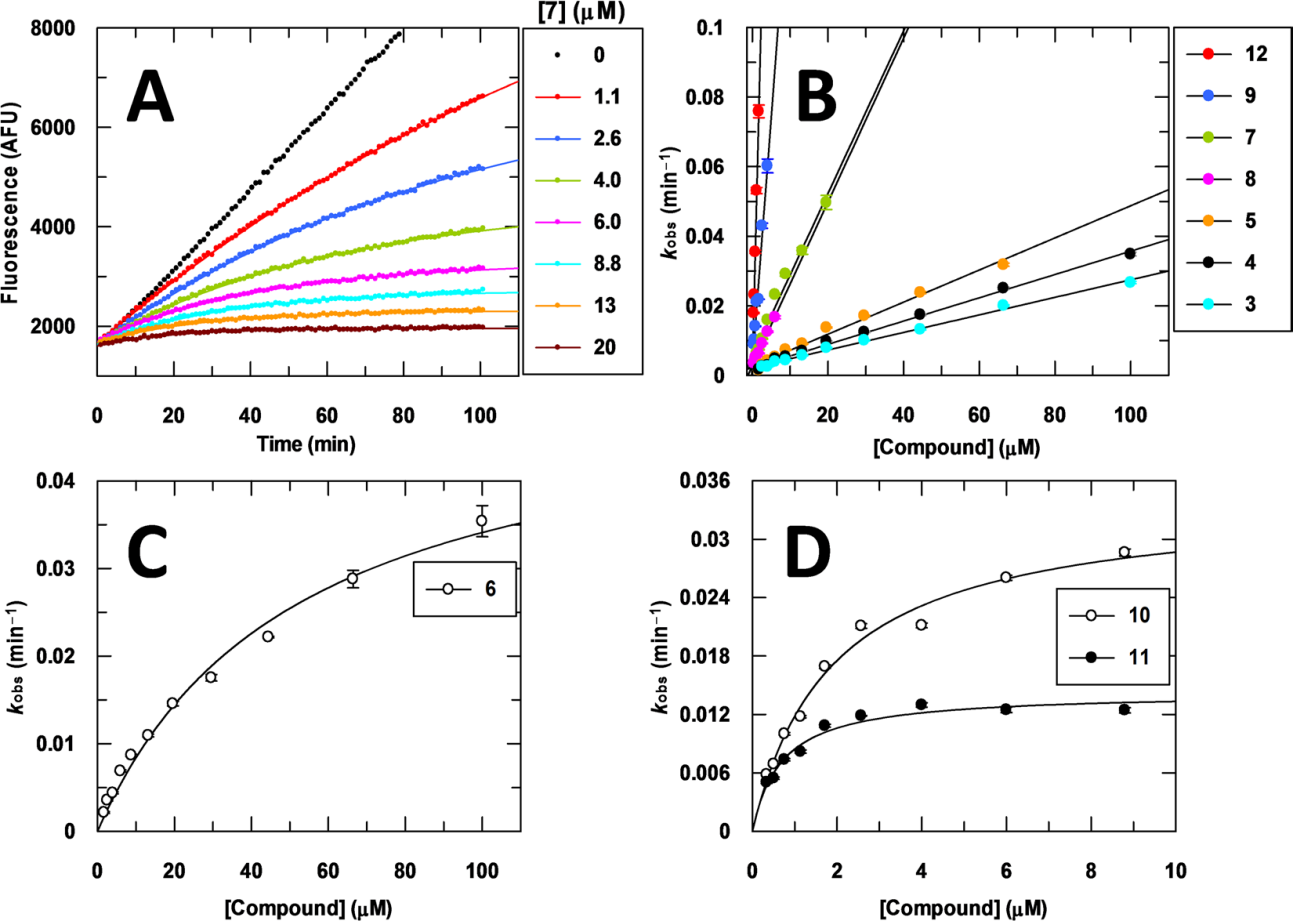
Progress curve analysis of inhibiton of Mtb-DprE1 by covalent inhibitors. (**A**) Representative time courses illustrating time dependent inhibition of Mtb-DprE1 by compound 7. (**B**–**D**) Dependence of *k*_obs_ on inhibitor concentration for a series of BTZs and BOZs.

We recently developed a fluorescence-based enzyme assay for Mtb-DprE1 that involves oxidation of farnesylphosphoryl-β-D-ribose (FPR) to farnesylphosphoryl-β-D-2′-keto-erythro-pentafuranose (FPX) as resazurin is reduced to the highly fluorescent resorufin^10^. Time courses are linear for approximately 100 min and reaction progress can be monitored continuously making this assay ideally suited for mechanism of inhibition studies by time dependent inhibitors. This assay made it possible to observe clear time dependent inhibition behaviour by the BTZs for the first time and, therefore, permitted a more detailed evaluation of the mechanism of inhibition of Mtb-DprE1 by this class of inhibitors (Fig. 2). Prior assays were monitored for only 2 min^9^, which was insufficient time to make these observations.

Progress curves were fitted to a single exponential equation to obtain the observed first order rate constant of inactivation, *k*_obs_ (Figs 2A and S1A and B, Supplementary Information)^18,19^. A wide range of inhibitor concentrations was used from 10 nM to 100 µM, never exceeding the measured solubility of each individual compound (Table 1, CLND solubility). The dependence of *k*_obs_ on compound concentration provides information concerning the number of steps involved in the mechanism of inactivation. Covalent inactivators that bind to enzymes with negligible affinity (e.g., covalent modification of catalytic cysteine residues by iodoacetamide) typically follow a single step binding mechanism as reflected by the linear dependence of *k*_obs_ on compound concentration (the slope of this relationship giving the second order rate constant for inactivation, *k*_inact_/*K*_i_). As the affinity increases, the slope of the *k*_obs_ versus inhibitor concentration relationship increases, and *k*_obs_ may show saturation behaviour if there is a change in the rate-limiting step from reversible binding at low inhibitor concentration to covalent chemistry at high inhibitor concentration.

Most BTZs and BOZs (3, 4, 5, 7, 8, 9, and 12) showed a linear dependence of *k*_obs_ on inhibitor concentration with a range of *k*_inact_/*K*_i_ values, and three (6, 10, and 11) showed a hyperbolic dependence (Table 1 and Fig. 2). Because the BTZs and BOZs are multi-step mechanism-based inhibitors involving different redox forms of the enzyme (E_OX_ and E_RED_), the *k*_inact_ and *K*_i_ constants are complex parameters that are composed of the rate constants of all the individual steps depicted in Fig. 1, which together will govern whether a linear or hyperbolic *k*_obs_ versus inhibitor concentration relationship will be observed in the experimentally attainable inhibitor concentration region. Differences in inhibitor structure can affect (1) the initial affinity of E_RED_ for the unactivated form of the inhibitor, (2) affinity of the resulting E_OX_:NO-BTZ complex, and (3) the rate constants of the individual chemical steps, the magnitude of which would depend on how optimal the inhibitor is positioned in the active site for inhibitor reduction and covalent bond formation to occur. Consequently, it is not possible to derive directly from *k*_inact_/*K*_i_ which of the individual rate constants and thus steps is responsible for the differences in rate of inactivation. Nevertheless, for covalent inhibitors, *k*_inact_/*K*_i_ is the most reliable parameter to use as a guide to optimize chemistry^18^; the higher the value, the more effective the compound is as a covalent inhibitor.

Comparing the first row of compounds in Table 1, substituting the piperidine group in 7 with a morpholine in 6, does not significantly compromise *k*_inact_/*K*_i_. Replacing the piperidine in 7 with dimethylpiperidine in 9 improves *k*_inact_/*K*_i_ by 6-fold. Introduction of a chlorine atom at the 7-position of the molecule in 11 results in an 8.7-fold increase in *k*_inact_/*K*_i_ as compared to 7. The improvements in *k*_inact_/*K*_i_ seem to track with lower MIC values in Mtb and *M. vaccae* (a non-infectious mycobacterial strain that is commonly used as a model system of Mtb), as would be expected.

We also explored the BOZ class of compounds using similar substitutions at the 2-position of the molecule as with the BTZs (middle row in Table 1). The *k*_inact_/*K*_i_ values of the BOZs (4, 3 and 5) are lower than the BTZ counterparts by 7-, 4- and 31-fold, respectively. Whilst the Mtb MIC value for 5 has increased 8-fold relative to 9, the MIC values for 4 and 3 did not change significantly in relation to 7 and 6. In *M. vaccae*, the MIC values for 4, 3 and 5 have also changed in relation to the BTZ counterparts but to a lesser degree than in Mtb. We have also obtained *k*_inact_/*K*_i_ values for BTZ043 (12)^4,20^ and PBTZ169 (13)^14,21^, the most potent BTZs reported to date. Whilst PBTZ169 was too potent to obtain a reliable *k*_inact_/*K*_i_ value due to assay tight-binding limit considerations, BTZ043 as expected gave the highest *k*_inact_/*K*_i_ value that could be reliably measured followed by 10, the BOZ equivalent of PBTZ169. Compared to the MIC of PBTZ169, the MIC of 10 is approximately 700-fold higher. Taken together, the results demonstrate that although the BOZs examined in this study cannot compete with the BTZ counterparts with respect to enzymatic and cellular potencies, substitution at the 1-position of the molecule with a heteroatom is tolerated and could be exploited in the future in conjunction with other changes in the molecule to improve potency.

**Figure 3:**
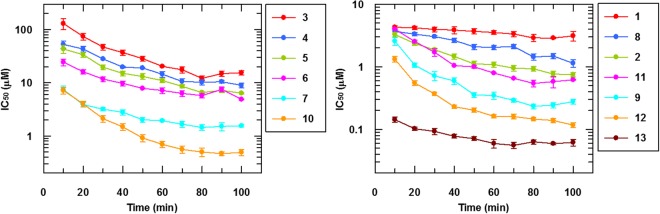
Time dependence of enzyme assay IC_50_ values. IC_50_ values of BTZs and BOZs as a function of time. All of the determined IC_50_ values decrease with the reaction time.

The slopes of the time courses of Figs 2 and S1A/B (Supplementary Information) were computed in 10 min increments to determine how the IC_50_ changes as a function of time (Fig. 3). All BTZ and BOZ IC_50_ values decrease with increasing reaction time as expected for time dependent inhibitors. The IC_50_ for an irreversible inhibitor should, in theory, reach a limiting value at prolonged incubation times (>5 × t_1/2_) and is expected to be equal to half the concentration of active enzyme that is present in the assay^18,19^. This appears to be approximately the case for the most potent compounds (12 and 13; Fig. 3) where the >5 × t_1/2_ criterion is satisfied best (the concentration of Mtb-DprE1 in these enzyme assays was 150 nM).

Significant loss of measured potency is often observed when going from a biochemical assay to a cellular assay. Most often, the loss results from poor compound permeability across cell membranes. This was indeed found for several noncovalent inhibitors of Mtb-DprE1, where the MIC values were approximately 100-fold higher than the IC_50_ values in the biochemical assay^10,22–26^. For covalent inhibitors such as BTZ043, however, the opposite appears to be true; the reported *M. smegmatis* and *M. tuberculosis* MIC values are approximately 1000-fold lower^4^ than the measured *M. smegmatis* DprE1 enzymatic IC_50_ value of 4.5 μM^9^. It should be noted that a direct comparison between enzymatic IC_50_ values and cellular MIC values for a given compound is not very meaningful because the two parameters measure different activities and the relationship between the two is very complex. However, the observed trends for a given series of compounds can be compared and discussed and our data in Fig. 3 provide an explanation for the above reversal. For 12 (same as BTZ043), the enzymatic DprE1 IC_50_ value decreases from approximately 1.5 μM after 10 min of reaction time to approximately 120 nM after 100 min of reaction time. Had the assay been monitored longer, in theory the IC_50_ value would have reached a limiting value of 75 nM (this is equal to half the concentration of enzyme in the assay)^18,19^. Owing to the very low turnover number of the enzyme^10^, the enzymatic assay is not sensitive enough to permit measurements to be made at low nanomolar enzyme concentration to experimentally demonstrate that the enzymatic IC_50_ values reach low nanomolar limiting values at prolonged incubation times.

Figure 3 also reveals the perhaps unexpected observation that the nitro-BTZ 12 inhibited Mtb-DprE1 much faster than its nitroso analogue, 8. The nitroso derivative does not need to be activated for covalent binding to Cys387, yet the time dependence of the inhibition is much slower than that of the nitro counterpart. As was proposed previously^7,9^, these data suggest that as 12 is reduced to 8, it immediately reacts with Cys387 in the active site with little chance of escape by dissociating from the enzyme. The activity loss of the nitroso-BTZ 8 compared to its congener nitro-BTZ 12 is almost certainly related to the reactivity of the nitroso group towards nucleophiles and redox partners although we did not look for respective side-products or alternative reaction partners.

### A new mechanism for covalent DprE1 modification: hydroxylamino-BTZs as suicide substrates

**Figure 4:**
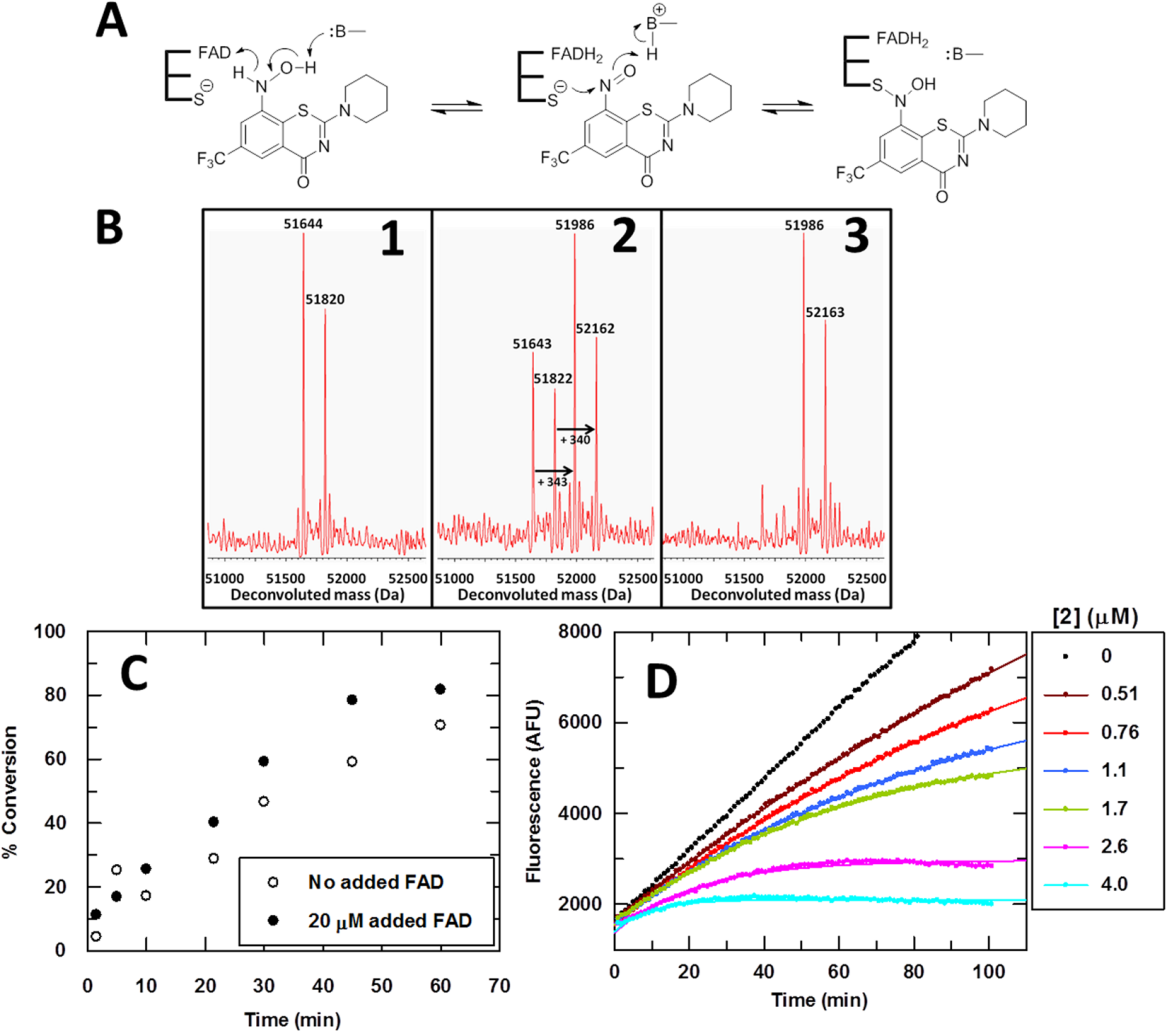
Covalent mode of inhibition of DprE1 by Hydroxylamino-BTZs. (**A**) Potential covalent mechanism of inhibition of Mtb-DprE1 by compound 2; (**B**) 1: Mass spectrum of Mtb-DprE1; 2: partial modification of Mtb-DprE1, 30 minutes after incubation with compound 2 and 20 μM FAD; 3: complete modification of Mtb-DprE1, 60 minutes after incubation with compound 2 and 20 μM FAD; (**C**) Time dependence of Mtb-DprE1 modification by 2 in the absence and presence of 20 μM added FAD; (**D**) Enzyme inhibition progress curves for compound 2.

The progress curves for 2 were also time dependent, which was not expected given that this hydroxylamine compound is the two-electron reduced form of the nitroso derivative, 1 (Fig. 4D). These data could be explained if 2 is an alternative substrate of Mtb-DprE1 that reduces E_ox_ to generate an E_RED_:1 complex, followed by covalent bond formation with Cys387 (Fig. 4A). Known antimycobacterial BTZs behave as “reverse substrates” of DprE1, meaning they have to be reduced, whereas DprE1 oxidizes its natural substrate. Our discovery shows that the physiological oxidative molecular mechanism may also be hijacked to inhibit DprE1. The mass spectra in Fig. 4B support our hypothesis.

**Figure 5:**
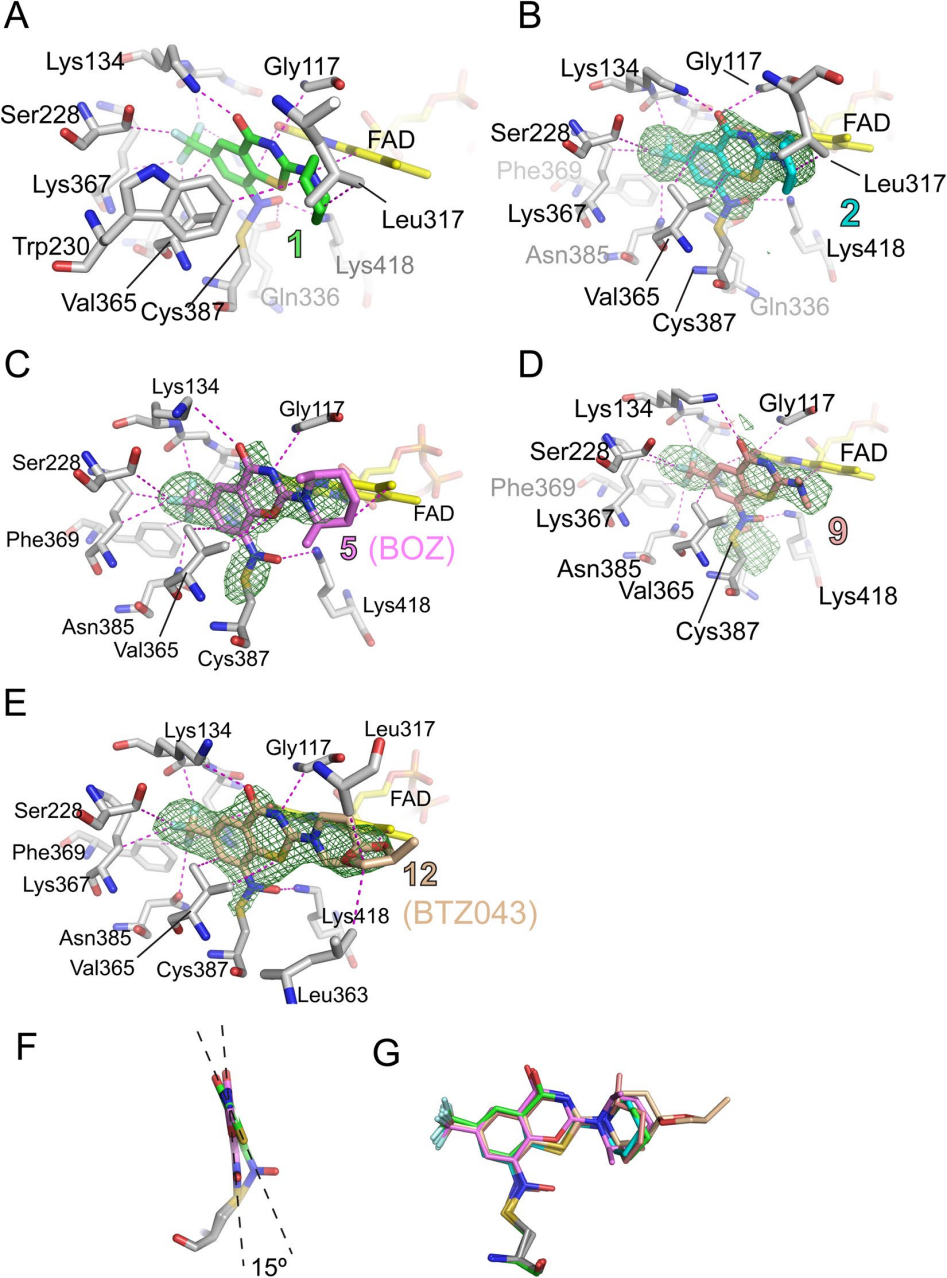
Contacts of BTZ and BOZ compounds with DprE1 and variation of inhibitor orientation. (**A**–**E**) Views of contacts (dashed lines in magenta) between BTZ, BOZ compounds with the active site of Mtb-DprE1. In (**A**), a 4 Å-distance cut-off was applied. Carbon atoms are colored in light grey (protein), yellow (FAD) and according to inhibitor identity. The unbiased Fo-Fc density (contour level 3σ) in panels B–E was calculated with model phases prior to incorporating the inhibitor in the coordinate set. In panel C, Trp230 was omitted for clarity. (**A**) Compound 1 (PDB entry 6HFW). (**B**) Compound 2 (PDB 6HFV). (**C**) Compound 5 (PDB 6HF0). (**D**) Compound 9 (PDB 6HF3). (**E**) Compound 12 (PDB 6HEZ, BTZ043). (**F**) Comparison of the orientation of the molecular planes of compounds 1 and 5. (**G**) Superimposing compounds 1, 2, 5, 9 and 12 after aligning the proteins according to secondary structure matching. Stick models are colored as in panels A–E.

We incubated 10 µM Mtb-DprE1 with 20 µM of the hydroxylamino-BTZ 2 *in the absence* of FPR and resazurin and monitored the intact mass of the enzyme by mass spectrometry (Fig. 4B). The mass spectrum of Mtb-DprE1 shows a 51644 Da species, which is consistent with the theoretical molecular weight of the *des*-Met form of the recombinant Mtb-DprE1 construct that we used. A second species (51820 Da) was also observed, which is 176 Da higher than the 51644 Da species and is likely due to an unknown covalent modification that occurred during expression of the recombinant enzyme in *E. coli*. The intensity of both species decreases with increasing incubation time, and two new species appear with molecular weights of 51986 Da and 52162 Da, which are 340–343 Da higher than the two starting species, consistent with the formation of the corresponding semimercaptal covalent derivatives (Fig. 4B,C). This hypothesis was further confirmed by X-ray data (Fig. 5B, see below) that clearly indicate a covalent bond to Cys387. The orientation of covalently-bound 2 in the active site of DprE1 is similar to the nitro and nitroso congeners. Although covalent modification of the enzyme required approximately one hour to reach completion using 20 μM of 2 under the conditions tested (Fig. 4C), the time frame is not dissimilar to the BTZs and BOZs. Addition of FAD did not significantly affect the kinetics of covalent modification. This is not surprising because the protein:FAD stoichiometry of the purified recombinant Mtb-DprE1 enzyme was determined to be 1:1, and under the high concentration of enzyme (10 μM) that was used in these experiments, the Mtb-DprE1 enzyme is expected to be saturated with the FAD that co-purified with the enzyme.

To exclude the possibility that DprE1 inhibition was caused by degradation products of 2, the stability of 2 in DMSO solution was checked by ^1^H-NMR and ESI-MS experiments. The compound was kept in aerated DMSO solution at room temperature for 13 days. No formation of the nitroso, nitro, or any other degradants was observed (data not shown).

Therefore, 2 is a true mechanism-based suicide substrate of Mtb-DprE1 because substrate (DPR or FPR) is not required for covalent modification of Cys387 to occur. This is in contrast to the nitro-BTZs where substrate, e.g., FPR, is required for inhibition to occur because activation of nitro-BTZs requires the presence of FADH_2_ in the catalytic center. As expected, in a control experiment using nitro-BTZ 12 in the absence of substrate, no change was observed in the mass spectrum of Mtb-DprE1 (data not shown). Very remarkably, 2 was very potent in the enzyme assay, surpassing its nitro analogue, 7 (Fig. 3 and Table 1).

Previous studies found BTZ046, which is the hydroxylamino analogue of BTZ043, to inhibit *M. smegmatis* DprE1 with an IC_50_ of 20 μM^9^. Time dependent inhibition was not observed. The MIC of BTZ046 against *M. smegmatis* of 1000 ng/ml or 2.4 μM is lower than the enzymatic IC_50_ value by approximately 8-fold, suggesting that the mechanism of action of BTZ046 at the cellular level may involve more than reversible binding to DprE1 with an affinity of approximately 20 μM. The same comment as above also applies here; direct comparison between enzymatic IC_50_ values and cellular MIC values for a given compound is not very meaningful because the two parameters measure different activities and the relationship between the two is very complex. Therefore, additional experiments would be needed to explore if BTZ046 indeed has an inhibitory mechanism with chemical reactions at the catalytic site of DprE1.

The DprE1 inhibition by the hydroxylamino-BTZ opens up new and exciting possibilities for DprE1 inhibition and thus antimycobacterial activity. An inhibitor that will take advantage of the enzyme’s regular physiological modus operandi – oxidation by FAD and covalent binding to Cys387 – will not have to wait for FADH_2_ to be formed through oxidation of another substrate. The hydroxylamino group is not common in drug substances because it forms strong hydrogen bonds with proteins, forms complexes with cations and impedes membrane permeability. This is perhaps the reason why the MIC values for 2 against *M. vaccae* (18.1 μM) and Mtb (>80 μM) are high in comparison with its nitro-BTZ analogue, 7 (low micromolar against both *M. vaccae* and Mtb). We are presently exploring other functional groups that may imitate the mechanism of inhibition that 2 displays.

### Mechanism of action of BTZs and BOZs: Mass spectrometric and crystallographic data

The accepted molecular mechanism of action of BTZs and other nitro-containing compounds, namely reduction of the nitro group to the nitroso oxidation state by enzyme-bound FADH_2_ followed by covalent bond formation of the nitroso intermediate with Cys387 (Fig. 1), is supported by several independent biochemical experiments^7^ as well as structural data^9,15^. However, alternative reaction pathways are also possible. For example, Tiwari *et al*. proposed a von-Richter-like mechanism involving nucleophilic substitution at the 7-position of the aromatic ring of the BTZ, mediated by a thiol/thiolate (e.g. Cys387) prior to formation of the reactive nitroso-BTZ^17^. In order to distinguish between these two possibilities, we synthesized nitro-BTZ 11, which has a readily substituted chlorine atom at the 7-position of the benzo moiety. If a von-Richter mechanism operates, loss of the chlorine atom should be detected by mass spectrometry.

We incubated Mtb-DprE1 with 11 in the presence of FPR and FAD and analyzed the protein product(s) by LC-MS (see Supplementary Information). A mass increase of 378 Da resulted, corresponding to the formation of the expected N-hydroxysulfenamide (semimercaptal) by reaction of the nitroso-BTZ with Cys387. This particular finding appears to rule out the operation of a von-Richter reaction because no species with a mass increase of 344 Da was observed i.e. 34 Da smaller than what was observed as required for substitution of the chlorine atom with a hydrogen atom. We used the nitroso-BTZ (1) and hydroxylamino-BTZ (2) as positive controls, which demonstrated that a +343 Da species is detected by mass spectrometry and therefore any trace of this species, if it occurred with 11, would have been observed (see Fig. 4 and Supplementary Information).

We obtained co-crystal structures of Mtb*-*DprE1 with BTZ compounds 1, 2, 9, and 12 and BOZ compound 5 (Fig. 5). Prior to crystallization, the enzyme was incubated with inhibitors and FPR^7^ in order to achieve covalent attachment as reported previously^9^. All structures were in space group *P*2_1_ with two DprE1-inhibitor complexes per asymmetric unit. Unbiased difference density maps, calculated with phases from structure models prior to incorporation of the ligand, demonstrated that all five compounds formed a covalent bond with the active site cysteine (Cys387) (Fig. 5B–E).

The orientation of the inhibitors in the active site is conserved among the compounds, owing to the semimercaptal link with Cys387, and due to steric constraints that prevent the otherwise possible 180° rotation about the C-N bond at the 8-position of the BTZ scaffold (consistent with reported data^9,14,27^). Accordingly, the invariant trifluoromethyl group is facing Lys134, Gly133, His132, Lys367 and Phe369 for all compounds. Despite the covalent bond to Cys387 and the spatial constraints imposed by the active site, there are still subtle variations with regards to the orientation of the BTZ double ring structure. For instance, the planes of the BTZ scaffold of compounds 1 and 5 enclose an angle of 15° (Fig. 5F), with the other three compounds in between these two extremes. The orientation of the hydroxyl of the semimercaptal bond is likewise not fixed, but always forming an H-bond interaction with Lys418. The substituents at the 2-position generally make only a few contacts with the protein and assume variable conformations (Fig. 5G). Table 2 provides a summary of non-covalent contacts (within a 4.0 Å cut-off distance) between the inhibitors and DprE1. The few non-conserved contacts are worth noting, such as Trp230, a highly conserved aromatic residue in the immediate vicinity of the active site, and Leu317, situated in the mobile loop (residues 315–329) that straddles the DprE1 active site. The apparent conservation of inhibitor-enzyme contacts is in marked contrast to the variation in inhibitory activity.

**Table 2:** Overview of non-covalent contacts between BTZ and BOZ inhibitors with DprE1.

Compound	12	5	9	1	2
Gly117	x	x	x	x	x
His132	x	x	x	x	x
Gly133	x	x	x	x	x
Lys134	x	x	x	x	x
Ser228	x	x	x	x	x
Trp230	—	x	—	x	—
Leu317	x	—	—	x	x
Gln336	x	—	x	x	x
Leu363	x	—	—	—	—
Val365	x	x	x	x	x
Lys367	x	x	x	x	x
Phe369	x	x	x	—	x
Asn385	x	x	x	x	x
Lys418	x	x	x	x	x
FAD	x	x	x	x	x

A shared feature of the structures of DprE1 determined to date^9,14,15,22,27^ is the two disordered loops (residues 268–285 and 315–330) that, based on geometric constraints, are presumed to straddle the active site. The complexes with BTZ and BOZ inhibitors induce partial ordering of the 315–330 loop in some cases (compounds 1, 2 and 12). In fact, in the structure of DprE1 bound to 12, some very weak density (below 1.0 sigma of the mean) can be discerned for residues 318 to 322. This weak density fails to define the conformation of the backbone sufficiently well to justify including residues 318–322 in the model. Still, this weak density indicates that, beyond residue 317, residues in the disordered loop do not contact the BTZ inhibitors in the covalent complex. The variation of inhibitory activity (in terms of IC_50_) with substituents at the 2-position contravenes the notion that only the covalent attachment to Cys387 and the conserved non-covalent contacts matter to the inhibitory mechanism. While the covalent complex is the end-point, it is preceded by non-covalent binding of the inhibitor to the ‘activated enzyme’, that is, when FAD is in the reduced state. In this process, non-conserved contacts with disordered loops may well matter.

Substitution of sulfur with oxygen at the 1-position (BOZ, compound 5) preserves the overall mode of binding (see Fig. 5C,D). With oxygen at the 1-position, the ring structure is more even, and we observe a small upward shift of position of the keto oxygen compared to the BTZ compounds. The conserved mode of binding in the covalent complex contrasts with the weaker inhibition (in terms of IC_50_) of DprE1 by BOZ compared to the BTZ sister compound as well as their differential MIC values (see assay section). Thus, the structures of the covalent complexes appear to represent ‘inhibitory endpoints’ that do not correlate with the different apparent IC_50_ values between compounds 5 and 9. Together, the data point to important differences in the process of the inhibitor first binding non-covalently to DprE1 complex en route to an eventual covalent link with Cys387.

The binding mode and positioning of 1 as shown in Fig. 5(C) was identical to the binding of the nitroso-BTZs that were formed from nitro-BTZs *in situ*.

The nitroso-BTZ 1 bound in the same way that CT325 did^15^, corroborating the ability of CT325 to serve as a BTZ substitute.

## Discussion

Since the discovery of the BTZs in 2009 and the identification of the DprE1 target^4^, several academic and industrial laboratories around the world have initiated drug discovery efforts to identify DprE1 leads. In addition to BTZ043 and PBTZ169, which are the most advanced nitro-BTZ derivatives^4,14^, many other classes of DprE1 inhibitors have been identified suggesting that DprE1 may be a “promiscuous” target^28^. However, the molecules seem to be specific inhibitors of DprE1 and no mammalian liabilities have been identified to date. DprE1 is, therefore, a highly tractable target stemming partly from its periplasmic localization^29^, partly from its high propensity to inhibition and partly from the lack of human DprE1 orthologues or homologues with significant sequence or structural similarity which would otherwise increase the chances of inhibiting mammalian enzymes and necessitate significant selectivity profiling efforts.

In order to understand the “promiscuity” of DprE1 and to help guide medicinal chemistry efforts to compound progression, mechanism of action studies are required such as the ones we carried out in this paper including detailed enzyme mechanism of inhibition studies, protein mass spectrometry and X-ray crystallography. Covalent strategies that target cysteine residues such as Cys387 of DprE1 are becoming increasingly popular in the pharmaceutical industry. Examples include irreversible as well as reversibly covalent warheads with prolonged and tunable residence times^30,31^. The availability of many potent reversible inhibitors of DprE1 suggests that engineering covalent warheads on these molecules to target Cys387 may be a promising future strategy to improve *in vivo* efficacy. This, however, requires structural information to inform on the locus of covalent attachment of the warhead on the molecules. Several crystal structures of DprE1 with inhibitors bound are available, most of which are covalent end-points including the five structures that we have determined in this study. To date, crystal structures with two classes of reversible inhibitors bound have been solved, TCA1^22^ and carboxyquinoxalines^26^. Additional structures with other classes of reversible inhibitors bound would open up a robust structure-based drug design approach to covalent strategies.

In conclusion, the mechanism of action details that we have developed in this study will help with the identification of the next generation DprE1 inhibitors. The BTZs are a promising antitubercular series of compounds. However, the high attrition rate of candidate molecules in clinical trials requires the continuous identification of new leads. Although the BOZs examined in this study are significantly worse than the BTZs, an MIC of 300 nM in Mtb has been achieved for the case of 10 and in conjunction with other changes in the molecule, further improvements are possible. Our studies also suggest that the development of new mechanism-based DprE1 inhibitors that take advantage of the oxidation cycle of DprE1 such as the hydroxylamino-BTZs is possible and may prove to be a promising new avenue to identifying the next generation DprE1 inhibitors. Although spontaneous mutation of Cys387 to Ser/Gly has been documented^4^ in Mtb and will lead to resistance to any new covalent compound that targets Cys387, any new TB therapy will almost certainly involve multiple drugs with different modes of action in order to minimize resistance as much as possible.

## Materials and Methods

Syntheses and characterization of the compounds, mass spectrometry experiments and additional tables and figures can be found in Supplementary Information.

### Chemicals

All chemicals were purchased from Sigma Aldrich, Alfa Aesar, VWR, Carl Roth, Fisher Scientific or Acros Organics and were used without further purification. All organic solvents, piperidine, 2,6-dimethylpiperidine, and DIPEA were distilled prior to use and stored with molsieve 3 Å. All solids were dried in a glass oven (Büchi TO-51, Büchi Labortechnik, Flawil, Switzerland) at 60 °C, 20 mbar for 60–120 min prior to use. Glassware for reactions under argon atmosphere were oven-dried at 100 °C for 2 h prior to use, evacuated and flushed with argon immediately. The process of evacuation and argon flushing was repeated for 3–5 times.

### Chromatography

Analytical thin layer chromatography (TLC) was performed on Merck silica gel 60 F_254_ precoated plates (Merck KGaA, Darmstadt, Germany) and visualized using an UV lamp (254 nm) or I_2_ stain. For flash chromatography, Merck silica gel 60 (40–63 µm) was suspended in an appropriate eluent, poured into glass columns and equilibrated with two column volumes of eluent. Compound was dissolved in 2 ml eluent and applied to the column or mixed with Celite 545 and acetone, the solvent evaporated and the residual celite-compound mixture applied as solid onto the flash column. Elution from flash chromatography columns was isocratic or using a gradient depending on the compound (see Supplementary Information).

### NMR spectrometry

NMR spectra were recorded on a Varian Inova 500 MHz (now Agilent Technologies, Böblingen, Germany) or Agilent Technologies VNMRS 400 MHz NMR spectrometers. Chemical shifts (δ) are reported in parts per million (ppm) relative to the residual protons of the deuterated solvent (chloroform δ 7.26, methanol δ 3.31, acetone δ 2.04, DMSO δ 2.49). NMR peak multiplicities abbreviations are as follows: s –singlet, bs – broad singlet, d – doublet, dd – double doublet, ddd – double doublet of doublet, dt – doublet of triplet, t – triplet, q – quartet, m – multiplet and coupling constants (J) are given in Hertz (Hz). NMR spectra were analyzed using mestrec23.

### Mass spectrometry

Electrospray ionization (ESI) mass spectra were recorded on a Thermo Finnigan Classic LCQ mass spectrometer (San Jose, California, USA). Samples were dissolved in an appropriate solvent and applied to the mass spectrometer via a syringe pump (injection volume 20 µl). The mass spectrometer settings were: capillary temperature 220 °C, voltage 4.5 kV, scanning range 50–2000 m/z. Electron impact (EI) mass spectra were recorded on an AMD 402 mass spctrometer (AMD Intectra GmbH, Harpstedt, Germany), with a medium ionization voltage of 70 eV.

### Elemental analysis

Elemental analysis was performed on a Leco CHNS-932 elemental analyzer (Leco Corporation, St. Joseph, Michigan, USA) or an Elementar Vario EL elemental analyzer (Elementar Analysensysteme GmbH, Hanau, Germany).

### Determination of compound solubility

Solubility of compounds was measured by Chemi-Luminescent Nitrogen Detection (CLND)^32^. 5 ml of 10 mM DMSO stock solutions of compounds were diluted to 100 ml with phosphate buffered saline (PBS), pH 7.4, equilibrated for 1 hour at room temperature and filtered through Millipore Multiscreen HTS-PCF filter plates (MSSL BPC) prior to analysis by CLND.

### Determination of Minimal Inhibitory Concentration (MIC)

MIC values for *M. tuberculosis* H37Rv and *M. vaccae* 10760 were determined as described previously^33,34^.

### DprE1 (Mtb) enzyme assays

The DprE1 enzyme assay has been described previously^10^. Compounds were dispensed in a black 384-well low-volume microplate (Greiner Bio-One, Stonehouse, UK; catalog no. 784076) using a Hewlett Packard HP D300 digital dispenser (Tecan Group Ltd.). Substrate mix (5 µl) containing 1 mM farnesylphosphoryl-β-D-ribose (FPR) and 50 µM resazurin in assay buffer (50 mM Hepes pH 7.5, 100 mM NaCl, 100 µM Tween-20 and 2 µM flavin adenine dinucleotide (FAD) was then added (both are final assay concentrations). The reactions were initiated by adding enzyme mix (5 µl) containing 150 nM Mtb-DprE1 in assay buffer, and resorufin formation was monitored spectrofluorimetrically (λ_ex_ = 530 nm, λ_em_ = 595 nm) using a Tecan Safire2 instrument (Tecan Group Ltd.).

Time courses were fitted to Eq. 1, where *F*_t_ and *F*_0_ are the fluorescence values at time, *t* and zero respectively, *k*_obs_ is the observed rate of inactivation and *A* is the change in fluorescence from zero to infinite time.

1 $${F}_{{\rm{t}}}=A\times (1-{e}^{-{k}_{{\rm{obs}}}t})+{F}_{0}$$

Where *k*_obs_ showed a linear dependence on the inhibitor concentration, the slope of such a relationship gave the second order rate constant of inactivation, *k*_inact_/*K*_i_. Where *k*_obs_ showed a hyperbolic dependence on the inhibitor concentration, *I*, the data were fitted to Eq. 2, where *k*_inact_ is the rate constant of inactivation and *K*_i_ is dissociation constant of the inhibitor from the enzyme. For these cases, *k*_inact_/*K*_i_ was calculated by dividing *k*_inact_ by *K*_i_.

2 $${k}_{{\rm{obs}}}=\frac{{k}_{{\rm{inact}}}[{\rm{I}}]}{{K}_{{\rm{i}}}+[{\rm{I}}]}$$

The IC_50_ values of Fig. 3 were obtained by fitting the dose-response curve data to Eq. 3, where *v* is the enzymatic rate of reaction at 10 min increments, *a* is the uninhibited value, *d* is the fully inhibited value, [I] is the inhibitor concentration, IC_50_ is the [I] that gives 1/2 × (*a*−*d*), and *h* is the Hill coefficient.

3 $$v=\frac{(a-d)}{1+{(\frac{[{\rm{I}}]}{{{\rm{IC}}}_{50}})}^{h}}+d$$

### Protein crystallography

#### Crystallization

To protein at 35 mg/ml in 20 mM Tris-HCl, pH 8.4 buffer containing 10% glycerol, FPR substrate, FAD and ligand were all added to 5 mM final concentration. After the reaction at room temperature in excess of 1 h, samples were left for approximately 54 h at 4 °C and then brought back to room temperature before fast desalting using the Pharmacia spin column (pre-equilibrated with buffer containing 5 mM ligand). For complexes with compounds 1 and 2, the protein was then crystallized in 1 + 1 µl sitting microbatch drops using a footprint screen of PPG400 and 100 mM Imidazole buffer, pH 7.4, PPG range from 24 to 34%- repeated three times. All of the 1 + 1 µl drops were covered in silicon oil and incubated at room temperature. Crystals were frozen directly from micro batch with addition of add cryoprotectant. For compounds 5, 9 and 12, purified DprE1 was incubated with inhibitor (50 μM), FPR (100 μM) and 1 mM MgCl_2_ at 37 °C for 2 hours. Crystallization experiments were set up in a 96-well plate format using a TTP Labtech liquid handling system, pipetting drops of 150 nl protein (~35 mg/ml) +150 nl reservoir solution. The composition of the reservoir solution was 50 mM imidazole pH 7.0–7.5, 28–31% v/v PPG 400. Crystals were mounted directly from the drop into nylon loops and flash frozen in liquid nitrogen.

#### Data collection and refinement

Data from single frozen crystals were collected on ID29 at the European Synchrotron Radiation Facility, Grenoble and beamline I03 at Diamond Light Source, Didcot, UK. Data were reduced using XDS and scaled using SCALA (CCP4) or XSCALE^35^. All crystals were in space group *P*2_1_ with 2 complexes of DprE1 bound to ligand in the ASU. The structures were phased by molecular replacement using a previously determined in house structure or PDB entry 4FDP^15^ as search models. Model building and refinement was performed using phenix.refine^36^, Coot^37^ and REFMAC^38^. Presence of ligand and covalent linkage to Cys387 was verified by unbiased Fo-Fc difference density maps phased using model coordinates prior to incorporation of the ligand in the model (Fig. 5). The covalency of the compounds was apparent by the contiguous density found between Cys387 and the nitrogen at the 8-position of the ligands. Data collection and refinement statistics are given in Table S3.

## Electronic supplementary material

Supplementary Information
