# Supplementary material for: Novel insight into the reaction of nitro, nitroso and hydroxylamino benzothiazinones and of benzoxacinones with Mycobacterium tuberculosis DprE1
Source: Sci Rep. 2018 Sep 7;8:13473. doi: 10.1038/s41598-018-31316-6 (PMC6128881; doi:10.1038/s41598-018-31316-6)
Supplement: Supplementary file 1 — Supplementary Information [file 41598_2018_31316_MOESM1_ESM.doc]

**Supplementary information to**

**Novel insight into the reaction of nitro, nitroso and hydroxylamino benzothiazinones and of benzoxacinones with Mycobacterium tuberculosis DprE1**

Adrian Richter^1^, Ines Rudolph^1^, Ute Möllmann^2^, Kerstin Voigt^2^, Chun-wa Chung^3^, Onkar M. P. Singh^3^, Michael Rees^3^, Alfonso Mendoza^4^, Robert Bates^4^, Lluís Ballell^4^, Sarah Batt^5^, Natacha Veerapen^5^, Klaus Fütterer^5^, Gurdyal Besra^5^, Argyrides Argyrou^3^*, and Peter Imming^1^*

^1^Institut für Pharmazie, Martin-Luther-Universität Halle-Wittenberg, Wolfgang-Langenbeck-Str. 4, 06120 Halle (Saale), Germany

^2^Leibniz-Institut für Naturstoff-Forschung und Infektionsbiologie - Hans-Knöll-Institut

Beutenbergstrasse 11a, 07745 Jena, Germany

^3^Platform Technology & Science, GlaxoSmithKline, Gunnels Wood Road, Stevenage, SG1 2NY, United Kingdom

^4^Diseases of the Developing World, Tres Cantos Medicines Development Campus, GlaxoSmithKline, Severo Ochoa 2, 28760 Tres Cantos Madrid, Spain

^5^School of Biosciences, University of Birmingham, Edgbaston Birmingham B15 2TT, United Kingdom

**Syntheses**

**General procedures:**

**General procedure I:** Synthesis of the benzoylchlorides

The corresponding benzoic acid was dissolved in toluene, 2 equivalents of SOCl_2_ added, the mixture refluxed for 2 h and subsequently the solvent evaporated under reduced pressure. The obtained benzoylchloride was used immediately for the next reaction step without further purification.

**General procedure II:** Synthesis of the benzothiazinone ring system

Dry KSCN was suspended in acetone and cooled to 5 °C. An equimolar amount of the corresponding benzoylchloride (synthesis general procedure I) was dissolved in acetone and added dropwise, subsequently the mixture was stirred for 2 h at 5 °C. Equimolar amounts of the corresponding amine were dissolved in acetone, added dropwise at approx. 10 °C and the mixture stirred for at 22 °C for 2 h. After evaporation of the solvent, the crude product was purified by flash chromatography.

**8-Nitro-2-(piperidin-1-yl)-6-(trifluoromethyl)-4H-1,3-benzothiazin-4-one (7)**

Synthesis of 2-chloro-3-nitro-5-(trifluoromethyl)benzoylchloride according to general procedure I from 100 mg (0.37 mmol) 2-chloro-3-nitro-5-(trifluoromethyl)benzoic acid.

Under argon atmosphere, 36 mg (0.37 mmol) KSCN were suspended in 5 ml acetone at 5 °C. 2-chloro-3-nitro-5-(trifluoromethyl)benzoylchloride was dissolved in 5 ml acetone and added dropwise, subsequently the mixture was stirred at 5 °C for 1.5 h. 36 µl (0.37 mmol) piperidine were dissolved in 5 ml acetone and added dropwise keeping the temperature at 5 °C. The mixture was then stirred at 5-10 °C for 2 h, the solvent evaporated and the crude product purified by flash chromatography twice (eluent TBME).The reaction yielded 0.016 g (12 %) of a pale yellow solid.

^1^H NMR (500 MHz, CDCl_3_) *δ* = 9.06 (d, 1H, Ar-H, *^4^J* = 2.1 Hz), 8.71 (d, 1H, Ar-H, *^4^J* = 2.1 Hz), 3.94 (m, 4H, CH_2_-N-CH_2_), 1.75 (m, 6H, CH_2_-CH_2_-CH_2_)

^13^C NMR (125 MHz, CDCl_3_) *δ* = 166.5, 161.5, 144.0, 134.3, 133.3 (q, *^3^J_C,F_=* 3.2 Hz), 129.5 (q, *^2^J_C,F_=* 35.4 Hz), 126.8, 125.9 (q, *^3^J_C,F_=* 3.7 Hz), 122.4 (q, *^1^J_C,F_=* 273.0 Hz), 47.9, 25.9, 24.3

MS (EI) 359 (M)

Elemental analysis calc. C 46.80 H 3.37 N 11.69 S 8.92

found C 46.91 H 3.38 N 11.55 S 9.54

**2-(2,6-Dimethylpiperidin-1-yl)-8-nitro-6-(trifluoromethyl)-4H-1,3-benzothiazin-4-one (9)**

Synthesis of 2-chloro-3-nitro-5-(trifluoromethyl)benzoylchloride according to general procedure I from 200 mg (0.74 mmol) 2-chloro-3-nitro-5-(trifluoromethyl)benzoic acid.

Under argon atmosphere, 72 mg (0.74 mmol) KSCN were suspended in 10 ml acetone at 5 °C. 2-chloro-3-nitro-5-(trifluoromethyl)benzoylchloride was dissolved in 5 ml acetone and added dropwise, subsequently the mixture was stirred at 5 °C for 1.5 h. 100 µl (0.74 mmol) 2,6-dimethylpiperidine were dissolved in 5 ml acetone and added dropwise keeping the temperature at 5 °C. The mixture was then stirred at 5-10 °C for 2 h and the solvent evaporated

Purification of crude product was achieved by flash chromatography twice (eluent DCM and chloroform:hexane 1:1 (V/V)). The fractions containing product were combined, the organic solvent evaporated and the residue recrystallized from hexane. The reaction yielded 0.098 g (34 %) of a pale yellow solid.

^1^H NMR (500 MHz, CDCl_3_) *δ* = 9.11 (d, 1H, Ar-H, *^4^J* = 2.2 Hz), 8.74 (d, 1H, Ar-H, *^4^J* = 2.2 Hz), 5.50 (bs, 1H, N-CH-CH_3_), 4.61 (bs, 1H, N-CH-CH_3_), 1.95 (m, 1H, CH_2_-CH_2_-CH_2_), 1.78 (m, 4H, CH_2_-CH_2_-CH_2_), 1.65 (m, 1H, CH_2_-CH_2_-CH_2_), 1.42 (m, 6H, 2 CH_3_)

^13^C NMR (100 MHz, CDCl_3_) *δ* = 166.2, 161.9, 144.0, 134.7, 133.2 (q, *^3^J_C,F_* = 3.5 Hz), 129.4 (q, *^2^J_C,F_* = 35.4 Hz), 126.9, 125.8 (q, *^3^J_C,F_* = 3.5 Hz), 122.4 (q, *^1^J_C,F_* = 273.3 Hz), 50.0, 49.1, 30.5, 29.8, 20.5, 19.9, 14.1

MS (ESI) *m/z* 388.20 [M+H]^+^

Elemental analysis calc. C 49.61 H 4.16 N 10.85 S 8.28

found C 49.99 H 3.99 N 10.84 S 7.76

**2-[4-(Cyclohexylmethyl)piperazin-1-yl]-8-nitro-6-(trifluoromethyl)-4H-1,3-benzothiazin-4-one (13)** Synthesis of 2-chloro-3-nitro-5-(trifluoromethyl)benzoylchloride according to general procedure I from 200 mg (0.74 mmol) 2-chloro-3-nitro-5-(trifluoromethyl)benzoic acid.

Under argon atmosphere, 72 mg (0.74 mmol) KSCN were suspended in 10 ml acetone at 5 °C. 2-chloro-3-nitro-5-(trifluoromethyl)benzoylchloride was dissolved in 5 ml acetone and added dropwise, subsequently the mixture was stirred at 5 °C for 1.5 h. 144 µl (0.74 mmol) 1-(cyclohexylmethyl)piperazine were dissolved in 5 ml acetone and added dropwise keeping the temperature at 5 °C. The mixture was then stirred at 5-10 °C for 2 h, the solvent evaporated and the crude product purified by flash chromatography twice (eluent TBME). The reaction yielded 0.121 g (35 %) of a pale yellow solid.

^1^H NMR (400 MHz, CDCl_3_) *δ* = 9.10 (d, 1H, Ar-H, *^4^J* = 2.1 Hz), 8.75 (d, 1H, Ar-H, *^4^J* = 2.1 Hz), 4.01 (m, 4H, CH_2_-N-CH_2_), 2.54 (m, 4H, CH_2_-N-CH_2_), 2.18 (d, 2H, N-CH_2_-CH, *^3^J* = 7.2 Hz), 1.74 (m, 5H, cyclohexyl), 1.49 (m, 1H, N-CH_2_-CH), 1.23 (m, 3H, cyclohexyl), 0.89 (m, 2H, cyclohexyl)

^13^C NMR (100 MHz, CDCl_3_) *δ* = 166.4, 162.0, 143.9, 134.1, 133.4 (q, *^3^J_C,F_* = 3.4 Hz), 129.7 (q, *^2^J_C,F_* = 35.5 Hz), 126.8, 126.0 (q, *^3^J_C,F_* = 3.8 Hz), 122.4 (q, *^1^J_C,F_* = 273.1 Hz), 65.1, 53.1, 46.6, 35.0, 31.7, 26.7, 26.0

MS (EI) 456 (M)

MS (ESI) *m/z* 457.2 [M+H]^+^

Elemental analysis calc. C 52.62 H 5.08 N 12.27 S 7.02

found C 52.20 H 4.92 N 11.82 S 6.54

**2-(morpholin-4-yl)-8-nitro-6-(trifluoromethyl)-4*H*-1,3-benzothiazin-4-one (6)** Synthesis of 2-chloro-3-nitro-5-(trifluoromethyl)benzoylchloride according to general procedure I from 809 mg (3.00 mmol) 2-chloro-3-nitro-5-(trifluoromethyl)benzoic acid.

The next steps were conducted under argon atmosphere according to general procedure II.

2-chloro-3-nitro-5-(trifluoromethyl)benzoylchloride was dissolved in 10 ml acetone and added dropwise to a solution of 291 mg (3.00 mmol) KSCN in 10 ml acetone. Upon complete addition, the mixture was heated to 40 °C for 5 min and then let cool again to rt. 261 µl (3.00 mmol) morpholine were dissolved in 10 ml acetone and added slowly. The mixture was stirred for another 30 min at rt, then heated to reflux for 2 min. After cooling, the solvent was evaporated under reduced pressure and the crude product was pre-purified by flash chromatography twice (eluent chloroform). The fractions containing product were combined, the solvent evaporated and the residue recrystallized from TBME. Yield 150 mg (13.8 %).

^1^H NMR (500 MHz, CDCl_3_) *δ* = 9.08 (d, *^4^J* = 1.5 Hz, 1H, Ar-H), 8.75 (d, *^4^J* = 1.5 Hz, 1H, Ar-H), 4.01 (m, 4H, CH_2_-N-CH_2_), 3.82 (m, 4H, CH_2_-O-CH_2_)

^13^C NMR (125 MHz, CDCl_3_) *δ* = 166.3, 162.6, 143.9, 133.7, 133.5 (q, *^3^J_C,F_=* 3.7 Hz), 129.9 (q, *^2^J_C,F_=* 35.9 Hz), 126.8, 126.1 (q, *^3^J_C,F_=* 3.7 Hz), 122.3 (q, *^1^J_C,F_=* 272. 5 Hz), 66.3 (bs, 2 CH_2_), 46.7 (bs, 2 CH_2_)

MS (EI) 361 (M)

Elemental analysis calc. C 43.22 H 2.79 N 11.63 S 8.87

found C 43.72 H 2.69 N 11.57 S 8.66

**8-(Hydroxyamino)-2-(piperidin-1-yl)-6-(trifluoromethyl)-4H-1,3-benzothiazin-4-one (2)** To a solution of 7 (0.050 g, 0.14 mmol) in THF (5 ml) a solution of NaH_2_PO_2_*H_2_O (0.080 g, 0.35 mmol) in water (5 ml) was added under protection atmosphere. After this, Pd/C catalyst was added to reaction mixture at 0 °C and the temperatur was slowly increased to 40 °C.

After 2 h an white preciptate occurred and the reaction was complete. The product was extracted with EtAc and after filtration through celite the organic layer was dried with MgSO_4_. For purification 2 was recrystalized from aceton. The reaction yielded 0.03 g (0.09 mmol, 63 %) of an white solid.

^1^H-NMR (400 MHz, DMSO-*d_6_*): δ = 8.95 (s, 1H, OH), 8.87 (s, 1H, NH), 7.90 (s, 1H, Ar-H), 7.51 (s, 1H, Ar-H), 3.81 (m, 4H, H-cyclohexyl), 1.65 (m, 6H, H-cyclohexyl)

^13^C-NMR (100 MHz, DMSO-*d_6_*): δ = 167.1, 160.3, 148.4, 128.4 (q, *^2^J_C,F_* = 32.4 Hz), 124.4 (q, *^1^J_C,F_* = 272.3 Hz), 123.4, 122.9, 116.8, 110.8, 47.3, 25.8, 24.2

MS (ESI) *m/z* 346.21 [M+H^+^], 368.13 [M+Na^+^]

HR MS *m/z* 346.0827 [M+H]^+^, calc. for [C_14_H_15_F_3_N_3_O_2_S]^+^ 346.0832

**8-Nitroso-2-(piperidin-1-yl)-6-(trifluoromethyl)-4H-1,3-benzothiazin-4-one (1)** To a solution of FeCl_3_*6H_2_O (0.129 g, 0.48 mmol) in water (20 ml) compound 2 (0.030 g, 0.09 mmol) dissolved in EtOH (40 ml), was added slowly.

After 5 h the reaction mixture was three times extracted with EtAc. The combined organic layers were dried over MgSO_4_ and the solvent was removed *in vacuo*. The residue was subjected to column chromatography (10 g SiO_2_, MTBE). The combined fractions were dried under vacuum to produce 21 mg (0.06 mmol, 70 %) of an intensive yellow solid.

^1^H-NMR (400 MHz, DMSO-*d_6_*): δ = 9.10 (d, *^4^J* = 2.0 Hz, 1H, Ar-H), 8.18 (d, *^4^J* = 2.0 Hz, 1H, Ar-H), 3.98 (m, 4H, H-cyclohexyl), 1.79 (m, 6H, H-cyclohexyl),

^13^C-NMR (100 MHz, DMSO-*d_6_*): δ = 166.0, 162.1, 158.9, 133.3, 132.8, 129.6 (q, *^2^J_C,F_* = 34.3 Hz), 125.3, 122.8 (q, *^1^J_C,F_* = 272.8 Hz), 121.9, 47.9, 25.9, 24.3

MS (ESI) *m/z* 344.12 [M+H^+^]

HR MS *m/z* 344.0673 [M+H]^+^, calc. for [C_14_H_13_F_3_N_3_O_2_S]^+^ 344.0675

**8-(Hydroxyamino)-2-{2-methyl-1,4-dioxa-8-azaspiro[4.5]decan-8-yl}-6-(trifluoromethyl)-4H-1,3-benzothiazin-4-one (14)** To a solution of 12 (0.020 g, 0.05 mmol) in THF (4 ml) a solution of NaH_2_PO_2_*H_2_O (0.028 g, 0.25 mmol) in water (4 ml) was added under protection atmosphere. After this, Pd/C catalyst was added to reaction mixture at 0 °C and the temperatur was slowly increased to 20 °C.

After 2 h the catalyst was removed by filtration through celite, the product was extracted with EtAc, the organic layers were dried over MgSO_4_ and the solvent was removed *in vacuo*. The reaction product, a white solid, was used without further purification.

MS (ESI) *m/z* 418.20 (M+H^+^), 440.15 (M+Na^+^)

**2-{2-Methyl-1,4-dioxa-8-azaspiro[4.5]decan-8-yl}-8-nitroso-6-(trifluoromethyl)-4H-1,3 benzothiazin-4-one (8)** To a solution of FeCl_3_*6H_2_O (0.060 g, 0.22 mmol) in water (4 ml) compound 14 (0.016 g, crude product) dissolved in EtOH (7 ml), was added slowly.

After 5 h the reaction mixture was three times extracted with EtAc. The combined organic layers were dried over MgSO_4_ and the solvent was removed *in vacuo*. The residue was subjected to column chromatography (10 g SiO_2_, MTBE). The combined fractions were dried under vacuum without any heating to produce 11 mg (0.03 mmol, 66 % over all yield) of an intensive yellow solid (8).

^1^H-NMR (400 MHz, CDCl_3_): δ = 9.11 (d, *^4^J* = 2.2 Hz, 1H, Ar-H), 8.20 (d, *^4^J* = 1.8 Hz, 1H, Ar-H), 4.30 (m, 1H, CH_2_), 4.15 (m, 4H, H-cyclohexyl), 4.13 (m, 1H, CH_2_), 3.51 (m, 1H, CH), 1.88 (m, 4H, H-cyclohexyl), 1.32 (d, *^3^J* = 6.1 Hz, 3H, CH_3_)

^13^C-NMR (100 MHz, CDCl_3_): δ = 166.1, 162.4, 158.8, 133.4, 132.6, 129.8 (q, *^2^J_C,F_* = 35.9 Hz), 125.1, 123.5 (q, *^1^J_C,F_* = 273.1 Hz), 122.1, 106.4, 72.5, 70.9, 44.8, 36.4, 35.2, 18.3

MS (ESI) *m/z* 416.21 [M+H^+^]

HR MS *m/z* 416.0883 [M+H]^+^, calc. for [C_17_H_17_F_3_N_3_O_4_S]^+^ 416.0886

**7-Chloro-8-nitro-2-(piperidin-1-yl)-6-(trifluoromethyl)-4*H*-1,3-benzothiazin-4-one (11)** Synthesis of 7-Chloro-8-nitro-2-(piperidin-1-yl)-6-(trifluoromethyl)-4H-1,3-benzothiazin-4-one by a new pathway covered by patent application.

^1^H NMR (400 MHz, CDCl_3_) *δ* 8.91 (s, 1H, Ar-H), 4.07 (bs, 2H, N-CH_2_), 3.70 (bs, 2H, N-CH_2_), 1.77 (m, 6H, CH_2_-CH_2_-CH_2_)

^13^C NMR (100 MHz, CDCl_3_) *δ* 165.7, 158.3, 146.9, 131.4, 130.5 (q, *^3^J_C,F_* = 5.3 Hz), 128.7 (q, *^2^J_C,F_* = 33.2 Hz), 128.4, 122.8, 121.6 (q, *^1^J_C,F_* = 274.3 Hz), 48.2 (bs, 2 CH_2_), 26.0 (bs, 2 CH_2_), 24.2

MS (EI) 393 (M)

Elemental analysis calc. C 42.70 H 2.82 N 10.67 S 8.14

found C 43.04 H 2.69 N 10.70 S 7.71

**2-(2,6-Dimethylpiperidin-1-yl)-8-nitro-6-(trifluoromethyl)-4H-1,3-benzoxazin-4-one** **(5)** Synthesis of 2-(2,6-Dimethylpiperidin-1-yl)-8-nitro-6-(trifluoromethyl)-4H-1,3-benzoxazin-4-one by a new pathway covered by patent application.

^1^H NMR (400 MHz, CDCl_3_) *δ* = 8.72 (d, 1H, Ar-H, *^4^J* = 2.2 Hz), 8.55 (d, 1H, Ar-H, *^4^J* = 2.3 Hz), 4.97 (bs, 1H, N-CH-CH_3_), 4.80 (bs, 1 H, N-CH-CH_3_), 1.89 (m, 1H, CH_2_-CH_2_-CH_2_), 1.75 (m, 4H, CH_2_-CH_2_-CH_2_), 1.60 (m, 1H, CH_2_-CH_2_-CH_2_), 1.39 (m, 6H, 2 CH_3_)

^13^C NMR (125 MHz, CDCl_3_) *δ* = 163.2, 155.5, 148.9, 136.3, 131.0 (q, *^3^J_C,F_* = 3.4 Hz), 127.3 (q, *^2^J_C,F_* = 35.5 Hz), 126.8 (q, *^3^J_C,F_* = 3.4 Hz), 122.3 (q, *^1^J_C,F_* = 273.5 Hz), 120.5, 48.4, 48.2, 30.0, 29.6, 21.1, 19.9, 13.4

MS (EI) 371 (M)

Elemental analysis calc. C 51.75 H 4.34 N 11.32

found C 51.78 H 4.08 N 11.11

**8-nitro-2-(piperidin-1-yl)-6-(trifluoromethyl)-4*H*-1,3-benzoxazin-4-one (4)** Synthesis of 8-nitro-2-(piperidin-1-yl)-6-(trifluoromethyl)-4H-1,3-benzoxazin-4-one by a new pathway covered by patent application.

^1^H NMR (400 MHz, CDCl_3_) *δ* = 8.72 (d, *^4^J* = 1.5 Hz, 1H, Ar-H), 8.57 (d, *^4^J* = 1.5 Hz, 1H, Ar-H), 3.90 (m, 4H, CH_2_-N-CH_2_), 1.75 (m, 6H, CH_2_-CH_2_-CH_2_)

^13^C NMR (125 MHz, CDCl_3_) *δ* 163.2, 155.1, 148.7, 136.2, 131.9, (q, *^3^J_C,F_* = 3.8 Hz), 127.4 (q, *^2^J_C,F_* = 35.5 Hz), 126.9 (q, *^3^J_C,F_* = 3.8 Hz), 122.2 (q, *^1^J_C,F_* = 273.5 Hz), 120.3, 46.7, 46.0, 25.9, 25.3, 24.0

MS (EI) 343 (M)

Elemental analysis calc. C 48.99 H 3.52 N 12.24

found C 49.00 H 3.40 N 12.28

**2-(morpholin-4-yl)-8-nitro-6-(trifluoromethyl)-4*H*-1,3-benzoxazin-4-one (3)** Synthesis of 2-(morpholin-4-yl)-8-nitro-6-(trifluoromethyl)-4H-1,3-benzoxazin-4-one by a new pathway covered by patent application.

^1^H NMR (400 MHz, CDCl_3_) *δ* = 8.71 (d, *^4^J* = 2.3 Hz, 1H, Ar-H), 8.59 (d, *^4^J* = 2.3 Hz, 1H, Ar-H), 3.93 (m, 4H, CH_2_-N-CH_2_), 3.83 (m, 4H, CH_2_-O-CH_2_)

^13^C NMR (100 MHz, CDCl_3_) *δ* = 162.9, 155.4, 148.5, 136.3, 131.1 (q, *^3^J_C,F_* = 3.4 Hz), 127.8 (q, *^2^J_C,F_* = 35.9 Hz), 127.1 (q, *^3^J_C,F_* = 3.4 Hz), 122.1 (q, *^1^J_C,F_* = 273.1 Hz), 120.3, 66.2, 66.1, 45.4, 45.0

MS (EI) 345 (M)

Elemental analysis calc. C 45.23 H 2.92 N 12.17

found C 44.87 H 2.64 N 11.79

**2-[4-(cyclohexylmethyl)piperazin-1-yl]-8-nitro-6-(trifluoromethyl)-4*H*-1,3-benzoxazin-4-one (10)** Synthesis of 2-[4-(cyclohexylmethyl)piperazin-1-yl]-8-nitro-6-(trifluoromethyl)-4H-1,3-benzoxazin-4-one by a new pathway covered by patent application.

^1^H NMR (400 MHz, CDCl_3_) *δ* = 8.72 (d, *^4^J* = 2.3 Hz, 1H, Ar-H), 8.58 (d, *^4^J* = 2.3 Hz, 1H, Ar-H), 3.93 (m, 4H, CH_2_-N-CH_2_), 2.54 (m, 4H, CH_2_-N-CH_2_), 2.19 (d, *^3^J* = 7.1 Hz, 2H, N-CH_2_-CH), 1.75 (m, 5H, cyclohexyl), 1.47 (m, 1H, N-CH_2_-CH), 1.24 (m, 3H, cyclohexyl), 0.89 (m, 2H, cyclohexyl)

^13^C NMR (125 MHz, CDCl_3_) *δ* = 163.0, 155.2, 148.6, 136.2, 131.0 (q, *^3^J_C,F_* = 3.5 Hz), 127.6 (q, *^2^J_C,F_* = 35.5 Hz), 127.0 (q, *^3^J_C,F_* = 3.3 Hz), 122.2 (q, *^1^J_C,F_* = 273.7 Hz), 120.3, 65.1, 53.0, 52.5, 45.0

MS (ESI) *m/z* 441.22 [M+H]^+^

HR MS *m/z* 441.1741 [M+H]*^+^*, calc. for [C_20_H_24_F_3_N_4_O_4_]^+^ 441.1744

**
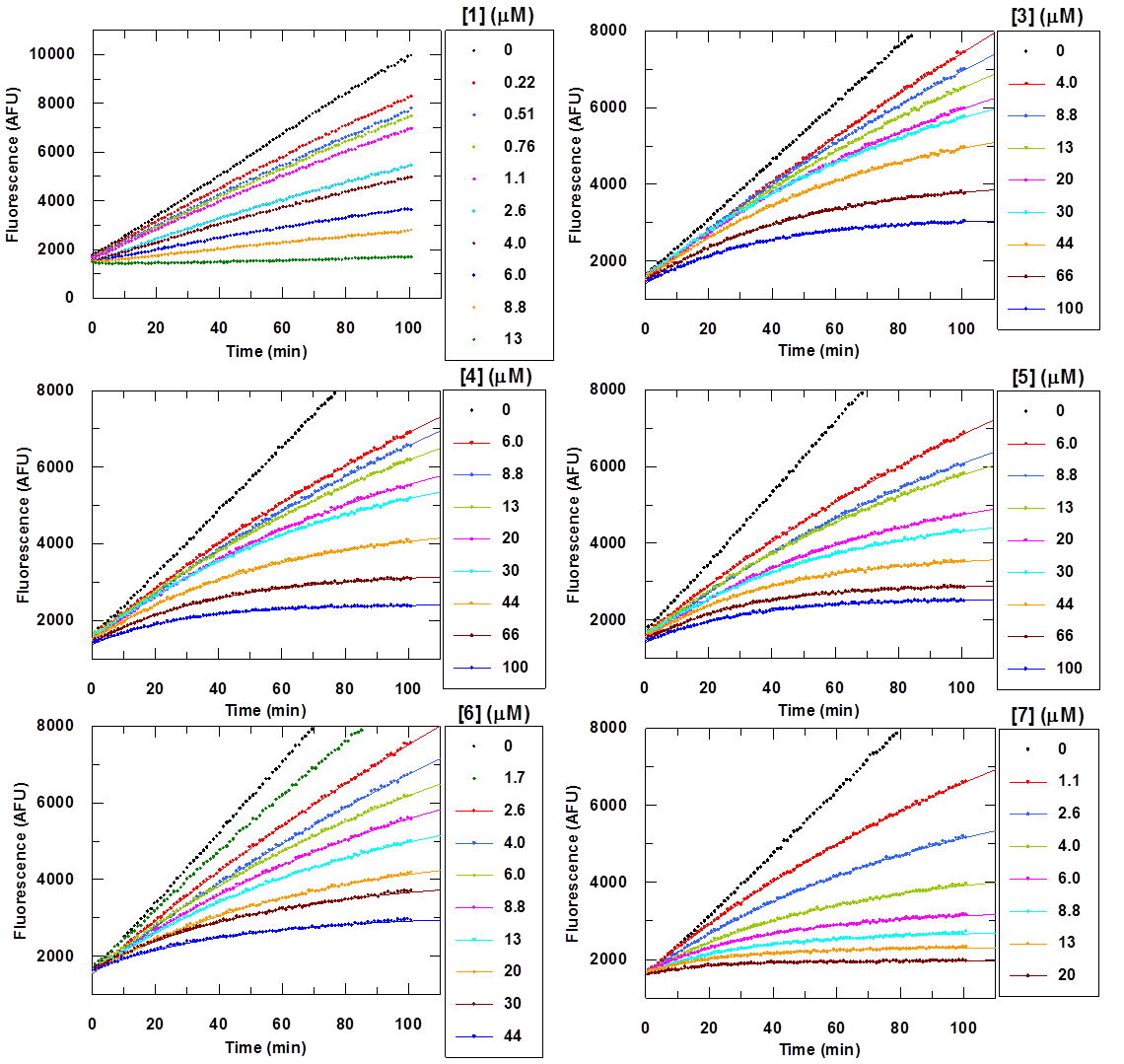
**

Figure S1A Inhibition curves

**
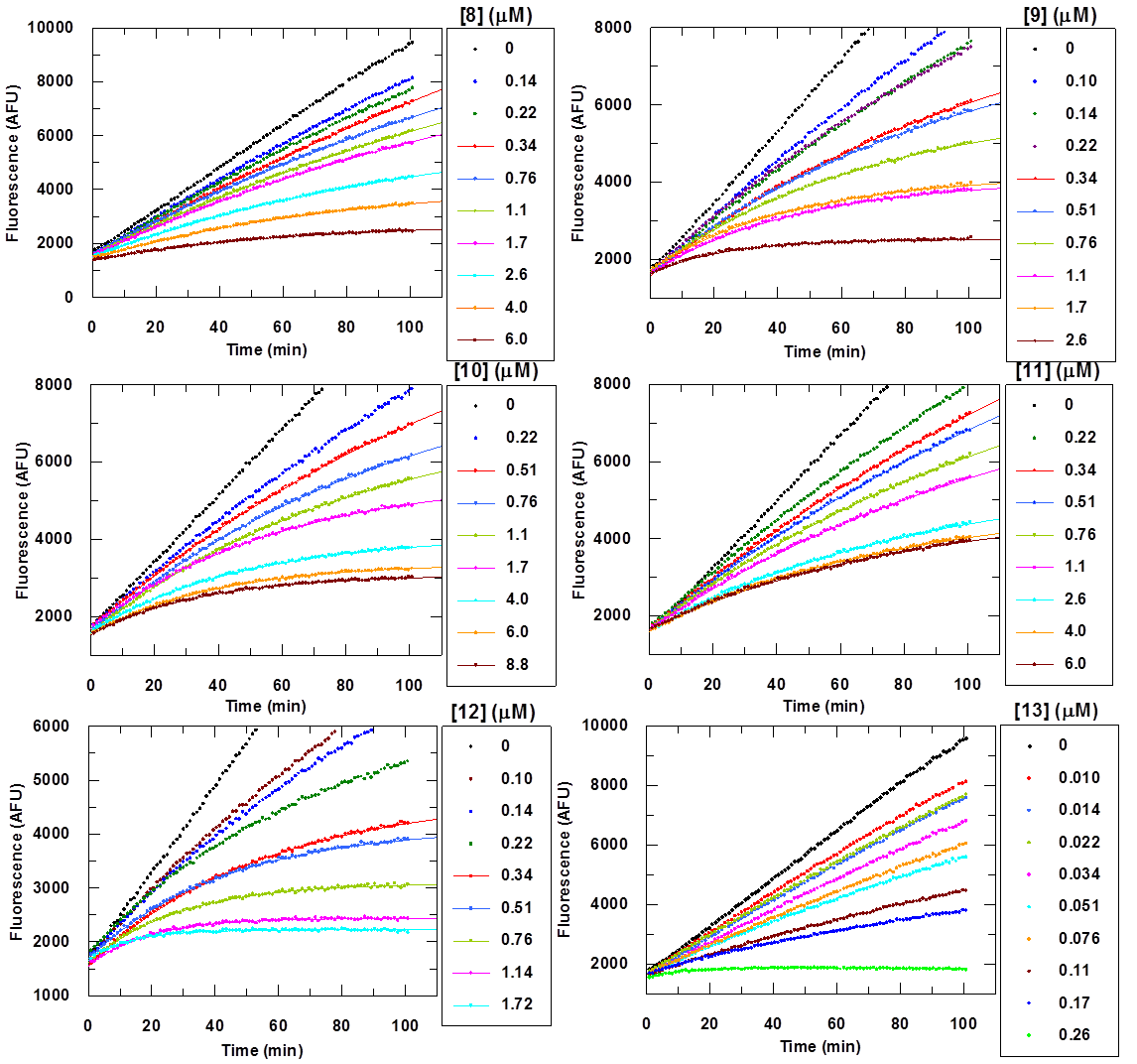
**

Figure S1B Inhibition curves

**Mass spectrometric data**

Protein incubation and data collection

To DprE1 at 35 mg/mL in 20 mM Tris-HCl, pH 8.4 buffer containing 10% glycerol, FPR substrate and ligand were added at 5 mM (condition A). For condition B additionally 5 mM of FAD was also added. After the reaction at room temperature for at least an hour, samples were left over the week-end at 4 °C and then brought back to room temperature before fast desalting using the Pharmacia spin column (pre-equilibrated with buffer containing 5mM ligand) These samples were diluted 4 µl to 40 µl with water. Protein Mass spectroscopy data was collected using two different instruments:

**Agilent 6224 ToF**

Data was collected using an Agilent 6224 ToF with 1200 liquid chromatography system. Samples were injected, 5ul at 0.1mg/ml protein, onto a 5 µm PLRP-S (Polymer Labs) 1 x 50 mm column. 1 x 50 mm.

LC Inlet conditions:

Buffer A 0.2% formic in water.

Buffer B 0.2% formic in acetonitrile.

Table S1 MS conditions

| Time | Flow | Presssure | %B |
| --- | --- | --- | --- |
| 0 | 0.5 | 350 | 10 |
| 0.5 | 0.5 | 350 | 30 |
| 5 | 0.5 | 350 | 55 |
| 5.7 | 0.5 | 350 | 60 |
| 5.71 | 0.5 | 350 | 100 |
| 6.2 | 0.5 | 350 | 100 |
| 6.21 | 0.5 | 350 | 10 |
| 8 | 0.5 | 350 | 10 |
| 8.1 | 0.5 | 350 | 10 |

MS conditions:

Ion polarity Positive.

Gas temp 340 °C

Gas flow 8 L/min

Nebulizer 60 psi

Data was collected and processed using Masshunter software. Raw data was deconvoluted to give intact protein mass.

This method was used to collect MS data of compounds 1, 11 and 13.

BTZs tested

**1** Mass: 343.3 Da

**11** Mass: 393.8 Da

**13** Mass: 456.5 Da


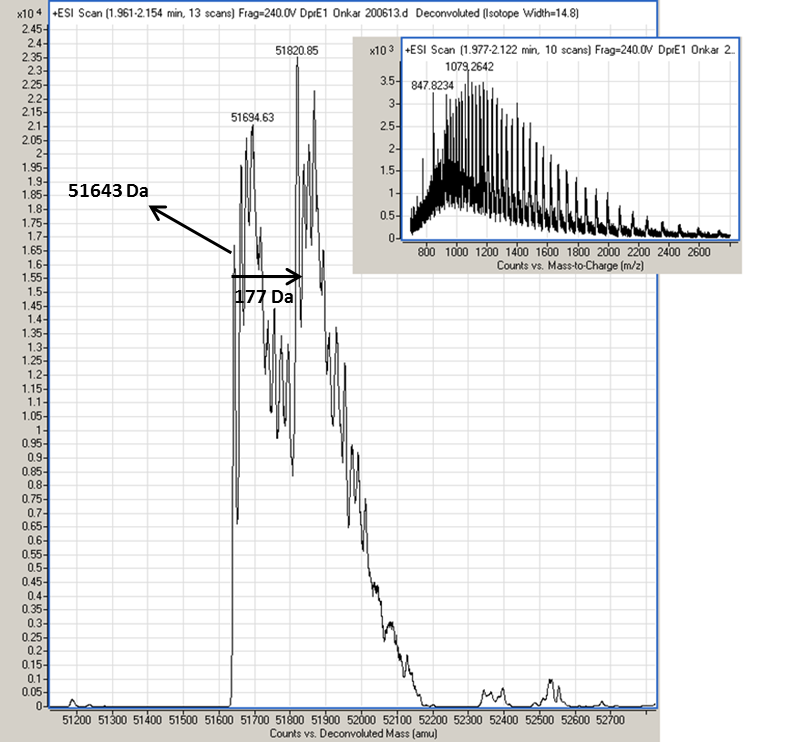


Figure S2 mass spectrum of the DprE1 without ligand


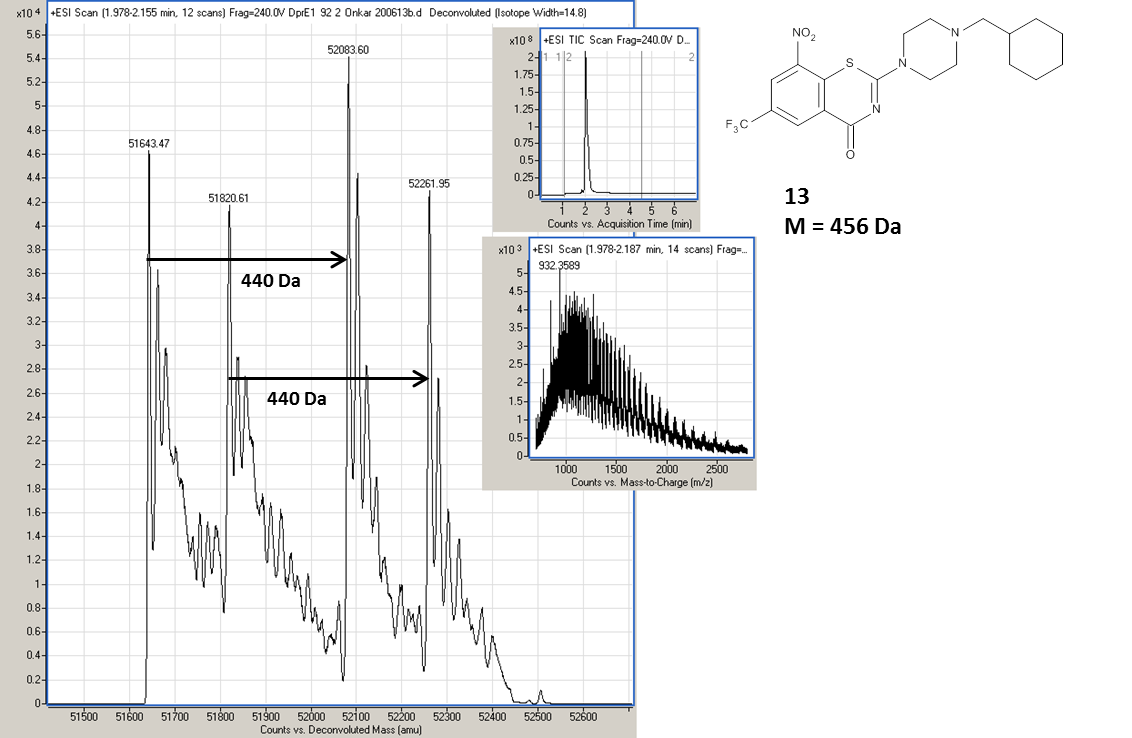


Figure S3 mass spectrum of DprE1 after incubation with 13 (condition A)


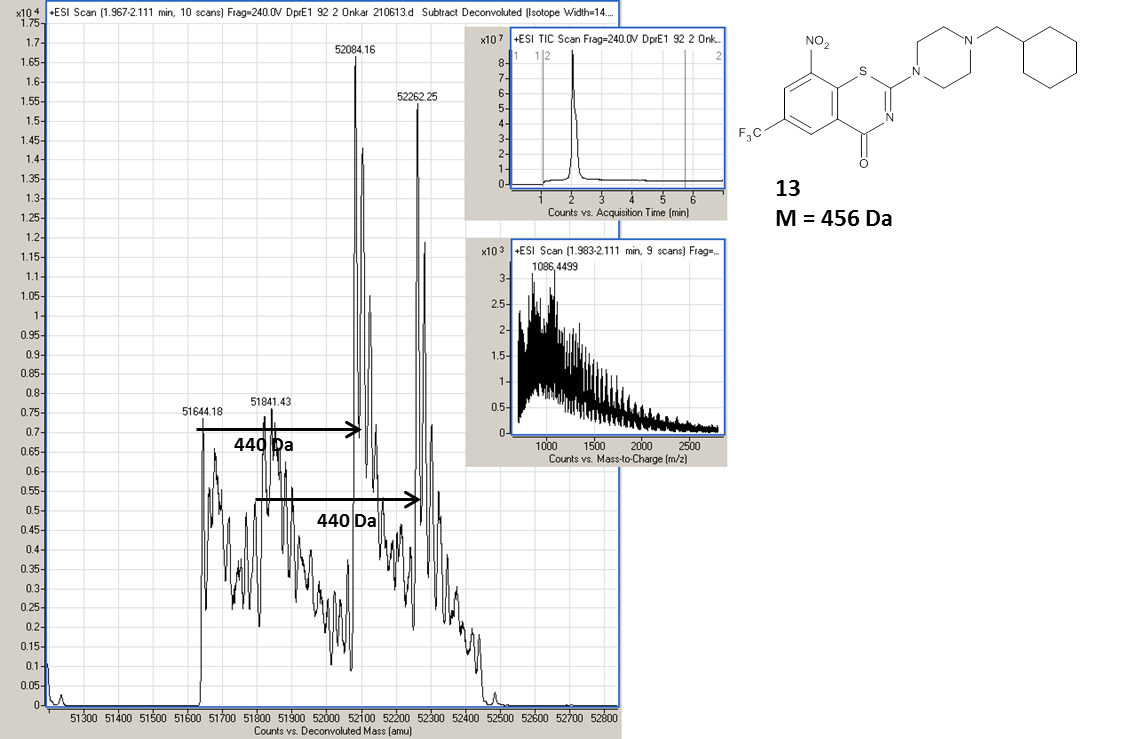


Figure S4 mass spectrum of DprE1 after incubation with 13 (condition B)


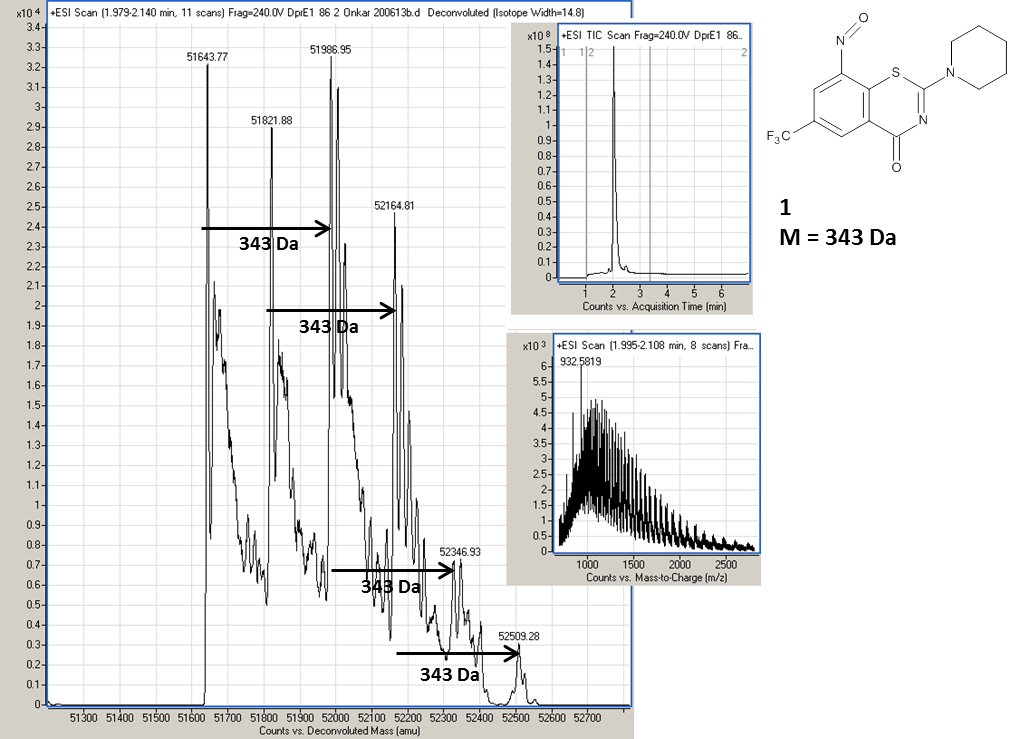


Figure S5 mass spectrum of DprE1 after incubation with 1 (condition A)


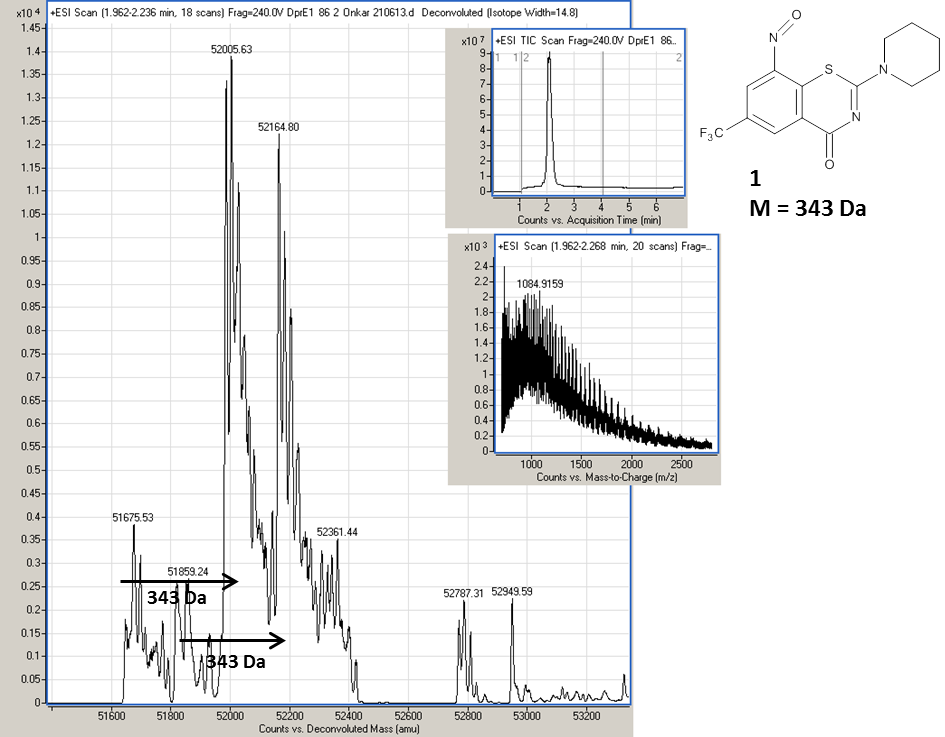


Figure S6 mass spectrum of DprE1 after incubation with 1 (condition B)


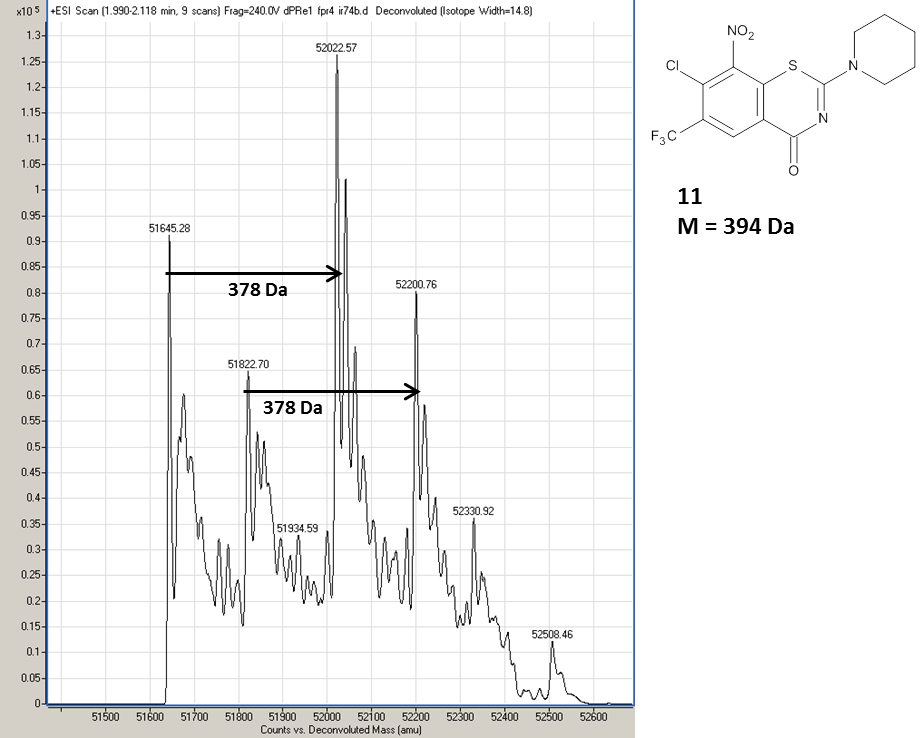


Figure S7 mass spectrum of DprE1 after incubation with 11 (condition A)


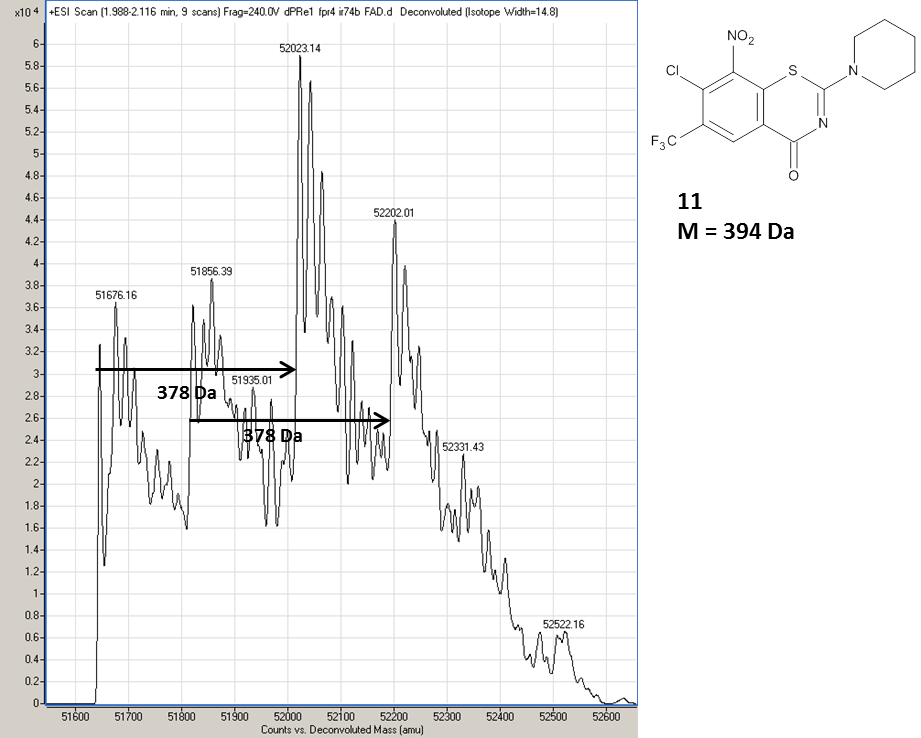


Figure S8 mass spectrum of DprE1 after incubation with 11 (condition B)

**Waters Q-ToF Ultima MS**

Samples were also injected (4 µl) onto a Waters Q-ToF Ultima MS using an Agilent 1100 liquid chromatography system fitted with a Zorbax Poroshell 300SB-C8 guard column (2.1 mm x 12.5 mm) cartridge running conditions below:

LC Inlet conditions:

Flow rate 0.5 ml/min

Column temperature 25 °C.

Buffer A, water 0.1% formic acid v/v.

Buffer B, acetonitrile 0.1% formic acid v/v.

Table S2 Gradient

| Time (min) | Buffer A % | Buffer B % | Flowrate ml/min |
| --- | --- | --- | --- |
| 0 | 95 | 5 | 0.5 |
| 0.5 | 95 | 5 | 0.5 |
| 2 | 10 | 90 | 0.5 |
| 3 | 10 | 90 | 0.5 |
| 3.1 | 95 | 5 | 0.5 |
| 4 | 95 | 5 | 0.5 |
| 5 | 95 | 5 | 0.5 |
| 5.1 | 50 | 50 | 0.5 |

MS Conditions:

ESI positive

Capillary 3.0 kV

Source Temp (°C) 100

Desolvation Temp (°C) 200

Cone Gas Flow (L/Hr) 100

Desolvation Gas Flow (L/Hr) 600

Data was collected using Masslynx software (v4.1) and deconvoluted using Maxent.

This method was used to collect the MS data of compound 7.

**Table S3**. X-ray diffraction data and refinement statistics.

| **X-ray diffraction data** |  |  |  |  |  |
| --- | --- | --- | --- | --- | --- |
| PDB entry | **6HFW** | **6HFV** | **6HF0** | **6HF3** | **6HEZ** |
| Compound | **1** | **2** | **5** | **9** | **12** |
| Beamline | ESRF, ID29 | ESRF, ID29 | Diamond I03 | Diamond I03 | Diamond I03 |
| Wavelength | 1.0725 | 1.0725 | 0.97631 | 0.97631 | 0.97631 |
| Space group | *P*2_1_ | *P*2_1_ | *P*2_1_ | *P*2_1_ | *P*2_1_ |
| Cell parameters a,b,c (Å), β | 78.3, 84.7, 80.9, 103.2º | 78.1, 84.4, 80.5, 103.0º | 78.6, 85.4, 80.4, 103.2º | 78.2, 84.1, 80.8, 103.4º | 78.5, 85.0, 80.5, 103.4 |
| Molecules in ASU | 2 | 2 | 2 | 2 | 2 |
| Resolution (Å) | 56.69 - 2.47 | 57.4 – 2.05 | 78.3 - 2.38 | 84.1 - 2.2 | 29.6 – 2.28 |
| High resolution shell (Å) | 2.60– 2.47 | 2.16 – 2.05 | 2.45 - 2.38 | 2.26– 2.20 | 2.34 – 2.28 |
| *R_merge_* (%)^1)^ | 6.0 (59.2) | 4.2 (51.4) | 4.8 (51.3) | 3.9 (61.0) | 4.2 (46.3) |
| Total observations | 103,392 | 214,517 | 212,901 | 193,532 | 174,538 |
| Unique reflections | 35,677 | 63,093 | 40,283 | 51,086 | 46,680 |
| *I/σ(I)* ^1)^ | 11.3(2.4) | 14.3 (2.6) | 16.3 (2.1) | 14.7 (2.4) | 14.5 (2.3) |
| Completeness (%)^1)^ | 96.8 (96.9) | 98.8 (98.5) | 97.1 (80.7) | 98.6 (97.0) | 99.0 (98.6) |
| Multiplicity^1)^ | 2.9(2.9) | 3.4 (3.5) | 5.3 (3.6) | 3.8 (3.9) | 3.7 (3.8) |
|  |  |  |  |  |  |
| **Refinement** |  |  |  |  |  |
| Resolution range | 56.69 – 2.47 | 57.0 – 2.05 | 78.3 - 2.38 | 76.1 – 2.2 | 29.6 – 2.30 |
| Unique reflections | 33,896 | 59,902 | 40,251 | 51,061 | 43167 |
| *R_cryst_ , R_free_* (%) | 17.0, 22.0 | 17.6, 20.8 | 21.2, 24.1 | 20.6, 23.8 | 19.8, 24.8 |
| No of non-hydrogen atoms | 6,863 | 7,019 | 6,511 | 6,562 | 6,627 |
| Protein + FAD | 6,589 | 6574 | 6,396 | 6,463 | 6,490 |
| Ligand | 46 | 46 | 42 | 38 | 56 |
| Solvent | 228 | 399 | 73 | 61 | 81 |
| RMSD bonds (Å) | 0.008 | 0.007 | 0.008 | 0.009 | 0.01 |
| RMDS angles (º) | 1.19 | 1.15 | 1.18 | 1.18 | 1.45 |
| B-factors |  |  |  |  |  |
| Wilson (Å^2^) | 66.8 | 41.3 | 53.0 | 47.3 | 45.6 |
| Average (Å^2^) | 64.2 | 72.2 | 67.8 | 62.4 | 62.6 |
| Protein + FAD (Å^2^) | 64.3 | 72.6 | 67.9 | 62.3 | 62.7 |
| Ligand (Å^2^) | 94.0 | 70.4 | 75.1 | 89.1 | 68.6 |
| Solvent (Å^2^) | 54.9 | 65.0 | 57.2 | 48.2 | 44.1 |
| RMSD B-factors (Å^2^) | 3.0 | 3.0 | 4.1 | 1.0 | 2.1 |
| Ramachandran plot^2)^ |  |  |  |  |  |
| Favoured region (%) | 98.4 | 98.9 | 97.6 | 98.2 | 97.6 |
| Allowed regions (%) | 1.6 | 1.1 | 2.4 | 1.4 | 2.3 |
| Disallowed (%) | 0.0 | 0.0 | 0 | 0.4 | 0.1 |

^1)^ Values in parenthesis refer to high resolution shell. ^2)^ Ramachandran plot statistics were calculated using Molprobity (Vincent B. Chen, W. Bryan Arendall III, Jeffrey J. Headd, Daniel A. Keedy, Robert M. Immormino, Gary J. Kapral, Laura W. Murray, Jane S. Richardson and David C. Richardson (2010) MolProbity: all-atom structure validation for macromolecular crystallography. Acta Crystallogr **D66**, 12-21)
